# Pyridyl‐Imidazopyridine Derivatives Displaying Submicromolar Activity Against *Trypanosoma cruzi*

**DOI:** 10.1002/cmdc.70377

**Published:** 2026-07-10

**Authors:** Ana Carolina Rocha Barreto, Kelly Lopes Figueira, Raquel Azevedo, Ketlym da Conceição, Ludmila de Ferreira de Almeida Fiuza, Luan dos Santos Vianna, Thiago Apolinário de Moraes, Wagner Alves de Souza Júdice, Maria de Nazaré Correia Soeiro, Jones Limberger

**Affiliations:** ^1^ Department of Chemistry Pontifical Catholic University of Rio de Janeiro Rio de Janeiro Rio de Janeiro Brazil; ^2^ Cell Biology Laboratory Oswaldo Cruz Institute, Oswaldo Cruz Foundation Rio de Janeiro Rio de Janeiro Brazil; ^3^ Interdisciplinary Center for Biochemical Research University of Mogi das Cruzes Mogi das Cruzes Brazil

**Keywords:** antitrypanosomal activity, Chagas disease, cruzain, imidazopyridines, molecular docking

## Abstract

Chagas disease, caused by the protozoan *Trypanosoma cruzi (T. cruzi)*, remains a major public health challenge due to the limited efficacy and high toxicity of current treatments. In this study, we describe the design and synthesis of 10 imidazo[1,2‐a]pyridine derivatives as potential antitrypanosomal agents. All compounds were evaluated in vitro against intracellular forms of *T. cruzi*, and the most active were further assessed for cytotoxicity and cruzain inhibition. Molecular docking studies were also conducted to investigate their binding modes in the enzyme active site. Some pyridyl‐substituted derivatives showed high potency, with submicromolar EC_50_ values. Among them, compound **5h** emerged as the most promising, with an EC_50_ of 0.16 μM, a selectivity index greater than 1250, and an IC_50_ of 10.6 μM against cruzain. Docking studies highlighted the importance of Cys25, Leu67, Met68, Ala133, and Glu205 in ligand recognition and suggested that chlorine substitution and pyridyl orientation influence cruzain binding. However, the discrepancy between the high cellular activity and the moderate cruzain inhibition, together with the weak inhibition observed for other active derivatives, suggests a likely multitarget mechanism of action. Overall, these findings highlight pyridyl‐imidazopyridines as promising scaffolds for the development of new anti‐*T. cruzi* agents.

## Graphical abstract

Previously reported pyridyl‐thiazoles and highly active imidazopyridines against *Trypanosoma cruzi* inspired the design of a new series of pyridyl‐substituted imidazopyridines. Among the synthesized compounds, **5h** emerged as the most promising derivative, displaying potent trypanocidal activity (EC_50_ = 0.16 μM), excellent selectivity (SI > 1250), and moderate cruzain inhibition (IC_50_ = 10.6 μM).

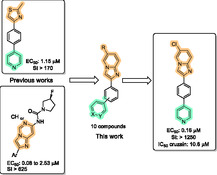

## 1 Introduction

Chagas disease (CD) is one of the most impactful neglected tropical diseases (NTDs) affecting approximately 7 million people worldwide. CD is endemic in 21 Latin American countries and accounts for more deaths than any other parasitic disease, affecting individuals regardless of age or gender [1, 3]. The infection progresses through two distinct clinical phases: acute and chronic. The acute phase occurs immediately after infection, and most cases remain asymptomatic or present only mild symptoms [4, 7]. In contrast, the chronic phase manifests years after the initial infection and can persist for a lifetime. While many patients remain symptom‐free, about 30%–40% develop severe cardiac and digestive complications, such as cardiomegaly, heart failure, arrhythmias, and megacolon or megaesophagus [4, 7]. Current treatment relies on chemotherapy with benznidazole (Bz) and nifurtimox (Nf), both developed in the 1960s and 1970s [2]. While these drugs are effective during the acute phase, their efficacy drops drastically in chronic patients. Furthermore, these medications are associated with high toxicity and severe side effects, including gastrointestinal distress and significant weight loss, leading to high rates of treatment discontinuation [8, 10].

Advances in Medicinal Chemistry and *Trypanosoma cruzi* (*T.* *cruzi)* biochemistry over the last decades have enabled the identification of novel molecular targets and accelerated the development of more selective and less toxic therapeutic candidates. Among the validated targets in *T. cruzi*, cruzain has attracted considerable attention [11]. As the major cysteine protease of the parasite, it is expressed throughout all stages of the life cycle and plays essential roles in parasite survival, differentiation and host–parasite interactions [12, 14]. Consequently, several classes of small molecules have been investigated as cruzain inhibitors, including N‐acylhydrazones, chalcones, ureas, thiazoles, and thiosemicarbazones [13, 15, 20]. Nevertheless, the complex biology of *T. cruzi* and the frequent occurrence of multitarget mechanisms among antiparasitic agents highlight the importance of exploring novel chemotypes and alternative modes of action.

**SCHEME 1:**
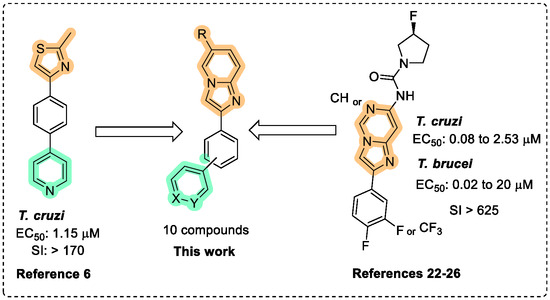
Design strategy for the imidazopyridine derivatives based on previously reported pyridylthiazoles active against *T. cruzi* [6] and imidazopyridine/imidazopyrimidine scaffolds active against both *T. cruzi* and *T. brucei* [22, 26].

Despite the well‐established pharmacological properties of imidazopyridines [21], including antitumor, anti‐inflammatory, antiviral, and antiparasitic activities, their application against *T. cruzi* remains relatively underexplored. Nevertheless, the limited studies reported to date have provided accumulating evidence that this heterocyclic scaffold represents a promising platform for the development of new antitrypanosomal agents, with several imidazopyridine derivatives displaying potent activity against trypanosomatids, including *T. cruzi* and *T. Brucei* (Scheme 1) [22, 26]. In particular, fluorophenyl‐substituted imidazopyridines exhibited remarkable potency against *T. cruzi*, with EC_50_ values below 100 nM and selectivity indices greater than 100 (Scheme 1) [22]. Likewise, other imidazopyridine‐containing compounds showed potent antiparasitic activity, low cytotoxicity, excellent metabolic stability, and favorable oral exposure in mice [23]. Although the mechanism of action of these compounds has not been fully elucidated, inhibition of the trypanosomal proteasome has been proposed for some derivatives based on their structural similarity to triazolopyrimidines [23]. However, the mechanism of action of antitrypanosomal imidazopyridines remains incompletely understood, and multiple molecular targets may contribute to their biological activity. In addition to the trypanosomal proteasome, cruzain has emerged as a relevant target for structurally diverse heterocyclic compounds displaying anti‐*T. cruzi* activity [27, 28]. Structurally related fused aza‐heterocycles [18], such as 1H‐pyrazolo[3,4‐b]pyridines, have shown potent trypanocidal activity and predicted interactions with cruzain in molecular modeling studies [29]. Likewise, quinazoline derivatives, which share a fused nitrogen‐containing aromatic framework with imidazopyridines, have been reported as potent competitive cruzain inhibitors and displayed significant trypanocidal activity [20]. Furthermore, pyridine‐ and thiazole‐containing derivatives were proposed to interact with cruzain based on mechanistic and in silico studies [19]. These findings suggest that nitrogen‐containing heteroaromatic scaffolds can engage biologically relevant targets beyond the proteasome and provided a rationale for evaluating cruzain as a potential target of the anti‐*T. cruzi* active imidazopyridines.

Additionally, our group previously reported thiazole derivatives bearing aryl and pyridyl substituents as anti‐*T. cruzi* agents [6]. Whereas methoxy‐ and fluoro‐substituted aryl derivatives displayed limited antiparasitic activity (0–11% inhibition at 10 µM), incorporation of a pyridine ring resulted in a substantial enhancement of potency, yielding compounds with EC_50_ values in the low micromolar range (1–2 µM) [6]. Based on these findings and on the promising antiparasitic profile previously reported for imidazopyridines [22, 26], we designed and synthesized 10 novel imidazo[1,2‐a]pyridine derivatives as structural analogs of our previously described thiazoles (Scheme 1), 6 of which contain pyridyl moieties. Their in vitro trypanocidal activity against *T. cruzi* was evaluated, and cruzain inhibition was investigated as a potential mechanism associated with their biological activity. While several compounds displayed potent antiparasitic effects, only a subset showed significant inhibition of cruzain, suggesting that additional targets may contribute to the observed trypanocidal activity. To further examine the interaction of the active compounds with this enzyme, molecular docking studies were performed using the cruzain‐active derivatives.

## 2 Results and Discussion

### 2.1 Design and Synthesis

The target imidazopyridines were initially conceived based on the previously reported activity of this class of compounds against *T cruzi* and *T. brucei* [22, 26]. In addition, this study aimed to investigate the effect of the isosteric replacement of the thiazole ring—present in compounds previously described as displaying EC_5_0 in the range of 1–2 μM against *T. Cruzi* (Scheme 1) [6]—with an imidazopyridine scaffold. Structural modifications included: (i) evaluation of the position of the nitrogen atom in the pyridine ring (3‐pyridyl vs. 4‐pyridyl); (ii) replacement of the pyridine moiety with aryl groups; (iii) variation of the substitution pattern on the central phenyl ring (*meta* or *para*); and (iv) introduction of substituents at the C‐6 position of the imidazopyridine core (H, Me, Cl). These modifications were explored to assess their impact on antiparasitic activity against *T. cruzi* and on the inhibitory activity toward the parasite cruzain.

**SCHEME 2:**
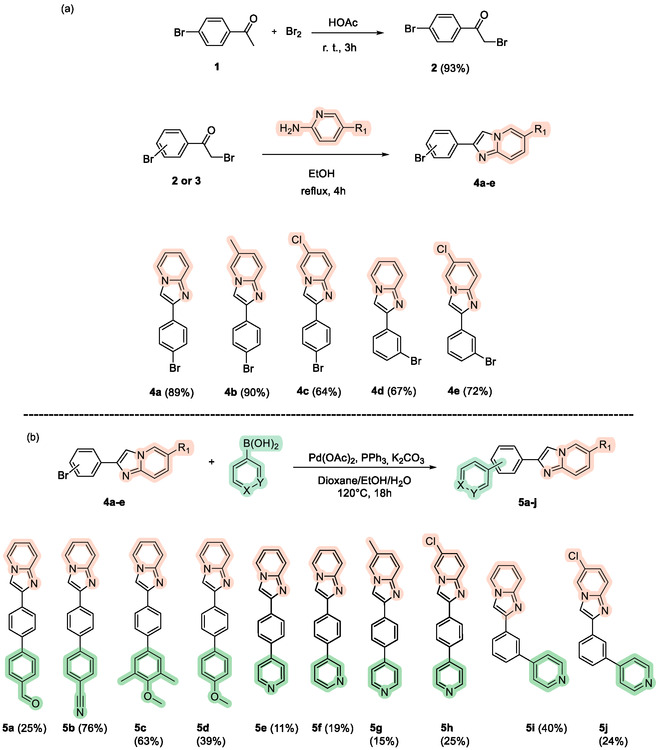
Syntheses and structures of imidazopyridines **5a–j**.

For the synthesis of these compounds, a three‐step route was applied. Initially, 4‐bromoacetophenone (**1**) was submitted to α‐bromination, yielding the intermediate (**2**) in 98% yield, whereas 2,3′‐dibromoacetophenone (**3**) was employed directly in its brominated form, bypassing the α‐bromination step. Intermediates **2** and **3** were then condensed with various substituted 2‐aminopyridines via Hantzsch reaction. This route was selected for its structural resemblance to the thiazole ring system, with reaction conditions tailored from the protocol described by Buu‐Hoï et al. [30]. These reactions afforded the intermediates **4a–e** with yields ranging from 64% to 90% (Scheme 2a). Finally, the brominated imidazopyridines intermediates **4a–e** were subjected to Suzuki cross‐coupling using a system based Pd(OAc)_2_, PPh_3_, K_2_CO_3_, and different aryl/pyridylboronic acids. This procedure afforded the target imidazopyridines **5a–j** with yields ranging from 11% to 76% (Scheme 2b).

### 2.2 Anti‐*T. cruzi* Activity and Cytotoxicity Evaluation

**FIGURE 1:**
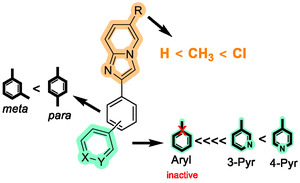
Structure–activity relationship elements for in vitro activity of the imidazopyridines.

The inhibitory effects of compounds **5a–j** against intracellular forms of *T. cruzi* were initially evaluated at fixed concentrations of 10 μM. Results were expressed as the percentage of infection reduction (Table 1) and Bz was included under identical conditions as a reference drug for comparison. Compounds **5a–d** presented a low activity (below 27% of reduction), suggesting a lack of activity of the methoxy, CN and COH substituents. On the other hand, compounds with a pyridine ring exhibited significant antiparasitic activity, with reductions ranging from 58% to 92% at the tested concentration (Table 1). The exception was compound **5f** which presented inhibition only slightly higher than the aryl‐substituited counterparts (**5a–d**). This initial finding supports the strategy of merging imidazopyridine and pyridyl units as a promise framework for developing antitrypanosomal molecules (Figure 1).

**TABLE 1:** In vitro activity of the synthesized compounds against intracellular *Trypanosoma cruzi* (Tulahuen strain expressing β‐galactosidase) after 96 h of treatment at 37°C (fixed concentration of 10 µM).

Compounds	Reduction on the infection index of the host cells,^a^ %
**5a**	≤21
**5b**	≤21
**5c**	≤27
**5d**	≤24
**5e**	87 ± 13
**5f**	34 ± 14
**5g**	57 ± 16
**5h**	92 ± 8
**5i**	58 ± 7
**5j**	59 ± 0.8
**Bz**	91 ± 0.7

Abbreviation: BZ, benznidazole.

a Mean ± standard deviation, two assays in triplicate.

The antiparasitic activity of pyridine‐substituted compounds **5e–j** was assessed as EC_50_ against the intracellular forms of *T. cruzi*, revealing compounds presenting highly potent profiles, with values in the submicromolar range (Table 2). The chlorinated analog **5h** emerged as the most active compound, with an EC_50_ of 0.16 ± 0.11 µM and a selectivity index (SI) > 1250, being approximately eightfold more potent than benznidazole (Bz, EC_50_ = 1.32 ± 0.02 µM; SI > 151) in this assay. Compounds **5g** and **5e** also displayed remarkable potency, with EC_50_ values of 0.29 ± 0.09 µM (SI > 690) and 0.37 ± 0.06 µM (SI > 541), respectively. In contrast, the 3‐pyridyl derivative **5f** exhibited a significantly reduced potency (EC_50_ = 4.43 ± 1.03 µM) and a markedly lower SI (SI = 18), highlighting the detrimental effect of the nitrogen atom positioned at the 3‐position of the pyridine ring (Figure 1). The superior activity observed for its corresponding 4‐pyridyl analog **5e** (Table 2, entry 1 vs. entry 2) reinforces the importance of nitrogen position for anti *T. cruzi* activity within this series. In addition to the position of the pyridine nitrogen, the data in Table 2 reveal other relevant structure–activity relationships for the anti‐*T. cruzi* activity. Comparison of the analog pairs **5h**/**5j** and **5e**/**5i** indicates that the para‐substitution pattern of the central ring resulted in more potent compounds (Figure 1). Moreover, analysis of derivatives **5e**, **5g**, and **5h** shows that substitution at the C‐6 position of the imidazopyridine core did not markedly affect the trypanocidal activity, as all three compounds displayed submicromolar EC_50_ values (Figure 1). Nevertheless, the chloro‐substituted derivative **5h** was slightly more active than its H‐ and Me‐substituted counterparts.

**TABLE 2:** In vitro antitrypanosomal activity (EC_50_), cytotoxicity (LC_50_), and selectivity indices (SI) of the imidazopyridine derivatives **5e–j** against intracellular *T. cruzi* amastigotes and L929 cells after 96 h of incubation at 37°C.

Compounds	EC_50_,^a^ µM	LC_50_,^a^ µM	SI
**5e**	0.37 ± 0.06	>200	>541
**5f**	4.43 ± 1.03	84 ± 4.0	18
**5g**	0.29 ± 0.09	>200	>690
**5h**	0.16 ± 0.11	>200	>1250
**5i**	10 ± 2	164 ± 60	16
**5j**	10 ± 3	>200	>20
**Bz**	1.32 ± 0.02	>200	>151

Abbreviations: BZ, benznidazole; EC_50_, half maximal effective concentration; LC_50_, lethal concentration at which 50% of the cells are killed; SI, Selectivity index.

a Mean ± standard deviation, two assays in triplicate.

In relation to cytotoxicity against murine L929 fibroblasts, compounds **5e**, **5g**, **5h,** and **5j** showed LC_50_ values higher than 200 µM, indicating low cytotoxicity and a favorable safety profile. Conversely, compound **5f** presented a lower LC_50_ value (84 ± 4.0 µM), which, combined with its higher EC_50_, resulted in a substantially reduced SI. Overall, the imidazopyridine scaffold associated with a 4‐pyridyl moiety demonstrated an optimal balance between potency and safety, yielding highly selective compounds, particularly derivative **5h**, which stands out as the lead candidate for further development.

### 2.3 Cruzain Inhibition

The inhibitory activity of compounds **5e–j** against cruzain was determined using a fluorogenic assay based on the hydrolysis of Z‐FR‐AMC. The IC_50_ values ranged from 10.6 to 291.6 µM (Table 3), revealing a marked dependence of enzymatic inhibition on the structural organization of the terminal heteroaromatic fragment. Cruzain, the major cysteine protease of *Trypanosoma cruzi*, is a validated molecular target for CD chemotherapy [31, 32]. Structurally, cruzain belongs to the papain‐like clan CA proteases and contains a catalytic Cys25–His162 dyad, with substrate recognition largely influenced by the S2 subsite [33]. The S2 pocket has been described as predominantly hydrophobic, although structurally organized and capable of accommodating aromatic moieties through shape complementarity and noncovalent interactions [33, 34].

**TABLE 3:** Cruzain inhibitory activity of the synthesized compounds **5e–j**, monitored by the hydrolysis of the fluorogenic substrate Z‐FR‐AMC (*λ*_ex_ = 360 nm; *λ*_em_ = 480 nm).

Compounds	IC_50_,^a^ µM
**5e**	47.3 ± 1.2
**5f**	291.6 ± 2.3
**5g**	N.d.
**5h**	10.6 ± 0.8
**5i**	195.6 ± 12.1
**5j**	166.6 ± 4.2

Abbreviations: IC_50_, half maximal inhibitory concentration; N.d., not determined; the intrinsic fluorescence of compound **5g** in the spectral range employed for IC_50_ determination interfered with the assay.

a Mean ± standard deviation, two assays in triplicate.

Within the evaluated series, compound **5h** was the most potent inhibitor (IC_50_ = 10.6 ± 0.8 µM), whereas **5f** displayed the lowest activity (IC_50_ = 291.6 ± 2.3 µM). Compound **5e** exhibited intermediate potency (47.3 ± 1.2 µM); the 4.46‐fold enhancement was observed from **5e** to **5h.** This improvement suggests that chlorine substitution favors a more productive interaction pattern within the cruzain active site, contributing to enhanced enzyme inhibition. Conversely, the 6.16‐fold reduction in potency from **5e** to **5f** highlights the detrimental effect of shifting the nitrogen atom from the 4‐position to the 3‐position of the terminal pyridyl ring. This decrease underscores the importance of nitrogen positioning for productive enzyme recognition, likely due to altered dipole orientation and suboptimal complementarity with the S2 subsite topology.

Positional isomerism in heteroaromatic systems is known to influence dipole orientation, electron density distribution, and overall molecular polarity [35]. In the context of enzyme inhibition, even small geometric or electronic alterations may translate into meaningful changes in binding free energy due to the highly structured environment of protease active sites [36]. The superior potency of **5h** relative to **5e**, combined with the reduced activity of the 3‐pyridyl isomer **5f**, demonstrates that both the substitution pattern on the imidazopyridine core and the nitrogen positioning within the terminal pyridyl ring are critical determinants of cruzain inhibition.

Compounds **5i** and **5j**, which bear the pyridyl substituent at the *meta*‐position of the central phenyl linker, exhibited IC_50_ values of 195.6 ± 12.1 and 166.6 ± 4.2 µM, respectively. These values correspond to approximately 4.1‐fold and 15.7‐fold lower potency compared to their para‐substituted analogs **5e** and **5h**, respectively. This consistent trend further supports the structural sensitivity of cruzain inhibition and suggests that the meta‐substitution pattern alters the spatial projection of the terminal heteroaromatic ring, compromising optimal S_2_ pocket occupancy.

The magnitude of potency variation observed across this relatively narrow chemical space (IC_50_ ranging from 10.6 to 291.6 µM) suggests that enzyme–ligand recognition is structurally discriminating rather than nonspecific. Similar sensitivity to aromatic substitution patterns has been reported for other classes of cruzain inhibitors, where S2 occupancy and spatial orientation of hydrophobic substituents significantly influence inhibitory potency [31, 34].

Collectively, these data indicates that cruzain inhibition in this imidazopyridine series is governed by three key structural features: (i) the presence of a halogen substituent at C‐6 of the imidazopyridine core, with chlorine providing substantial enhancement; (ii) the 4‐pyridyl substitution pattern, which is strongly preferred over the 3‐pyridyl isomer; and (iii) the *para*‐substitution on the central phenyl linker, which provides superior activity compared to *meta*‐substitution. Within the derivatives evaluated, compound **5h** represents the most favorable configuration and provides a rational starting point for further structure‐based optimization efforts aimed at improving cruzain inhibitory potency.

It is important to mention, however, that a pronounced discrepancy was observed between the remarkable antiparasitic potency of compounds **5h** and **5e** in cellular assays, including submicromolar EC_50_ values, and their limited or nearly negligible inhibition of cruzain. These findings strongly suggest that cruzain is not the primary target of these pyridyl‐substituted imidazopyridines, but rather a secondary target potentially contributing to their biological profile. Consequently, the antitrypanosomal activity of this series is more likely associated with a multitarget mechanism of action.

### 2.4 Molecular Docking

To elucidate the binding modes and explain the inhibitory variations among the imidazopyridine series, molecular docking simulations were carried out within the cruzain active site (PDB: 1ME4). The computational method was validated by redocking the cocrystallized ligand, achieving an RMSD of 0.9574 Å with the GoldScore scoring function, which was subsequently employed for all compounds. The resulting scores for each compound are summarized in Table S1.

All compounds interact with key residues in the catalytic site of cruzain, including Ala133, Cys25, Leu67, and Met68, although the nature and extent of these interactions vary among the derivatives (Table S2). The S2 subsite, which is predominantly hydrophobic and plays a crucial role in substrate recognition, accommodates the aromatic moieties of the ligands through shape complementarity and noncovalent interactions [33, 34].

**FIGURE 2:**
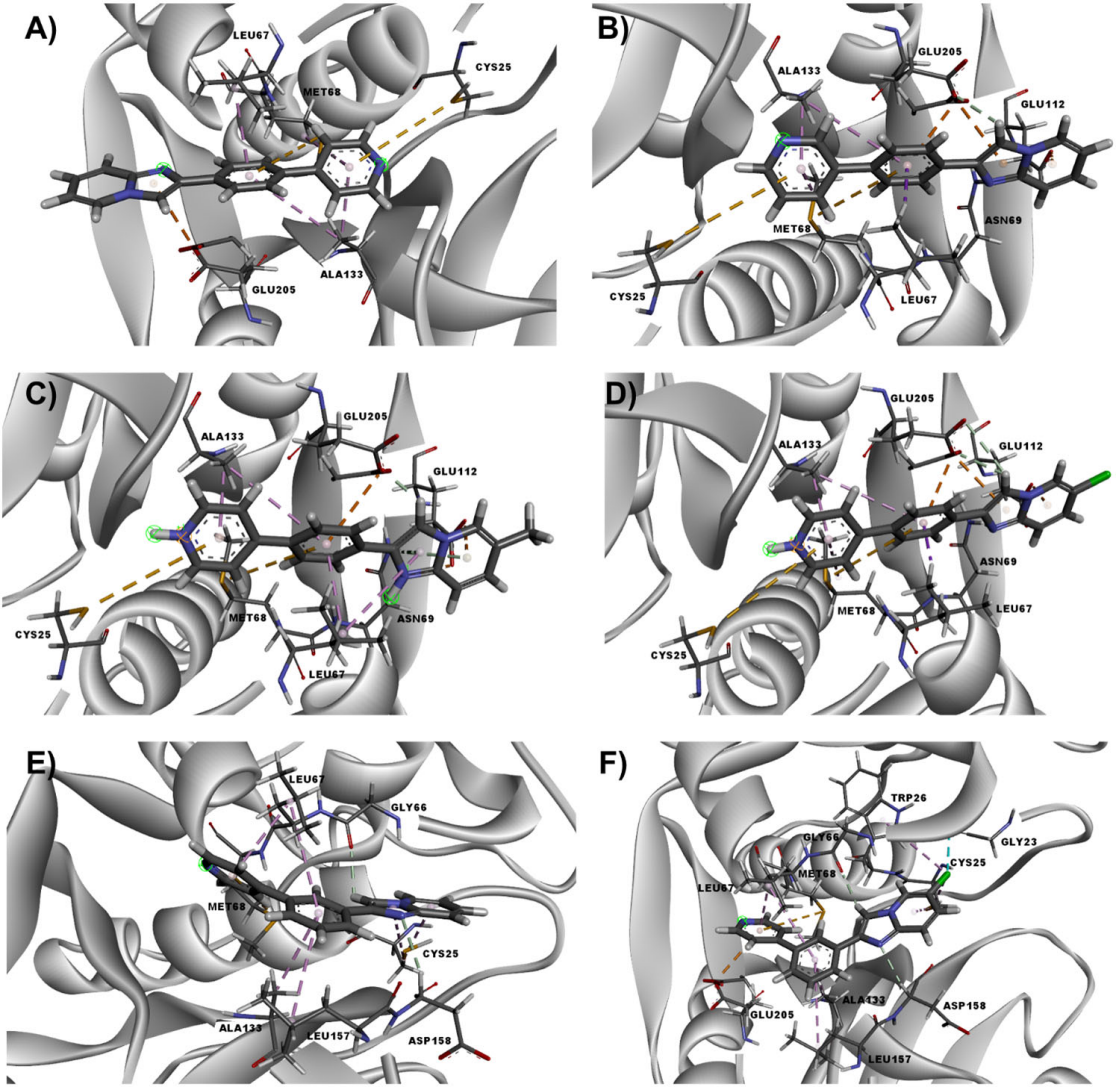
The best docking conformations for compounds **5e** (A), **5f** (B), **5g** (C), **5h** (D), **5i** (E), and **5j** (F) using the GoldScore function on GOLD.

Compounds **5i** (Figure 2E) and **5j** (Figure 2F), which bear the pyridyl substituent at the *meta*‐position of the central phenyl linker, exhibit relatively higher docking scores and establish a greater number of interactions of diverse natures compared to their *para*‐substituted analogs. Nevertheless, they exhibit substantially higher IC_50_ values (195.6 ± 12.1 and 166.6 ± 4.2 μM, respectively, Table 3), suggesting that the additional interactions predicted by docking do not necessarily translate into improved enzyme inhibition. This apparent discrepancy can be rationalized by careful examination of the binding poses. Although the *para*‐to‐*meta* substitution increased the number and diversity of predicted interactions, it also induced a distinct binding orientation within the active site. In particular, compounds **5e** and **5h** performed π–sulfur interactions with the catalytic residue Cys25, while compounds **5i** and **5j** primarily established π–alkyl and alkyl contacts with the same residue. Given the recognized importance of Cys25 for cruzain catalysis, these findings suggest that the nature of the interaction with this residue may be more relevant to inhibitory activity than the overall number of contacts predicted by docking. Furthermore, while additional interactions with peripheral residues such as Gly23, Gly66, Leu157, and Asp158 may contribute to overall binding affinity, they do not compensate for the suboptimal positioning relative to the catalytic machinery. This observation highlights the distinction between binding affinity and inhibitory potency.

When comparing the *para*‐pyridyl series, compounds **5e** and **5f** displayed very similar docking scores and interaction profiles, both establishing contacts with key residues within the active site. In addition, compound **5f** exhibited supplementary interactions with Asn69 and Glu112. However, these differences were not reflected in the biological assays, where **5f** (291.6 ± 2.3 μM) showed substantially lower inhibitory activity than **5e** (47.3 ± 1.2 μM). This result suggests that the positional change of the pyridine nitrogen affects molecular recognition in a manner that is not fully captured by the docking calculations, highlighting the limitations of relying exclusively on predicted interaction patterns and scoring functions to explain inhibitory potency.

The 4‐pyridyl substitution pattern appears to provide optimal dipole orientation and spatial projection for complementarity with the S2 subsite topology. Positional isomerism in heteroaromatic systems is known to influence dipole orientation, electron density distribution, and overall molecular polarity [35], and even small geometric or electronic alterations may translate into meaningful changes in binding free energy due to the highly structured environment of protease active sites [36].

The chlorinated derivative **5h** (Figure 2D), the most potent cruzain inhibitor in this series (IC_50_ = 10.6 ± 0.8 μM), displayed a favorable binding mode characterized by a well‐balanced network of hydrophobic, electrostatic, and polar interactions within the enzyme active site. Compared with the parent compound **5e**, the introduction of the chlorine atom resulted in a substantial increase in inhibitory potency, highlighting the positive contribution of this substituent to cruzain inhibition. Notably, **5h** established π–sulfur interactions with the catalytic residue Cys25 and Met68, in addition to contacts with Leu67, Ala133, Asn69, Glu112, and Glu205, contributing to the stabilization of the enzyme–ligand complex. Although compound **5j** also contains a chlorine atom, its activity was considerably lower than that of **5h**. This observation indicates that the beneficial effect of chlorine is strongly dependent on the overall binding geometry. The *para*‐oriented pyridyl ring in **5h** appears to support a more favorable interaction pattern, whereas the *meta*‐substitution in **5j** alters the ligand orientation and interaction network within the active site, resulting in reduced inhibitory activity despite its slightly higher docking score (Table S2).

A notable observation from this study is the lack of direct correlation between cellular antitrypanosomal activity (EC_50_) and cruzain inhibition (IC_50_). While compound **5h** demonstrates both potent cellular activity (EC_50_ = 0.16 μM) and the strongest cruzain inhibition within the series (IC_50_ = 10.6 μM), compounds **5e** and **5g** exhibit submicromolar cellular potencies (EC_50_ = 0.37 and 0.29 μM, respectively) despite displaying only moderate (**5e**, IC_50_ = 47.3 μM) or undetermined (**5g**) cruzain inhibition. This differential profile suggests a potential multitarget mechanism of action for the imidazopyridine series. While **5h** demonstrates that good cruzain inhibition correlates with exceptional cellular activity, the contrasting profiles of **5e** and **5g** suggest that additional molecular targets may be involved in the overall biological activity of this chemical series. Potential candidates include other parasitic proteases (e.g., cathepsin B‐like proteases), enzymes involved in ergosterol biosynthesis, or components of the trypanosomal proteasome, as previously suggested for structurally related imidazopyridines [22, 23].

## 3 Conclusion

In summary, a series of imidazo[1,2‐a]pyridine derivatives bearing phenyl–pyridyl substituents was evaluated for their antitrypanosomal potential. Several compounds displayed pronounced activity against intracellular *T. cruzi* amastigotes, with derivatives **5e**, **5g**, and **5h** exhibiting submicromolar potency. Among them, compound **5h** emerged as the most potent derivative (EC_50_ = 0.16 μM), showing approximately eightfold higher activity than benznidazole in these conditions. Most compounds also showed low cytotoxicity toward mammalian host cells (LC_50_ > 200 μM), indicating a favorable selectivity profile. Evaluation of cruzain inhibition revealed that **5h** acts as a moderate enzyme inhibitor (IC_50_ = 10.6 μM). In contrast, the weak cruzain inhibition observed for compounds **5e** and **5g**, despite their submicromolar cellular potency, suggests the involvement of additional molecular targets and a multitarget mode of action. Molecular docking studies of the cruzain‐active derivatives indicated key interactions with residues in the catalytic site, including Ala133, Cys25, Leu67, Met68, and Glu205. Combined with the enzymatic data, these findings suggest that cruzain inhibition is modulated by the position of the pyridyl nitrogen, chlorine substitution, and the substitution pattern of the central phenyl linker, highlighting key structural features associated with enhanced activity. Overall, these findings identify chlorinated imidazopyridines bearing a 4‐pyridyl moiety as promising scaffolds for the development of new antitrypanosomal agents.

## 4 Experimental

### 4.1 Materials and Methods

All solvents and reagents were purchased from commercial suppliers and used as received, unless otherwise indicated. Solvents employed in Suzuki reactions were deaerated using nitrogen flow. Compounds **2** and **4a–e** were synthesized following previously reported procedure [6, 30, 37]. Novel compounds were characterized via ^1^H and ^13^C NMR (Bruker Advance III HD 400 MHz spectrometer), FTIR (Bruker INVENIO, using ATR mode), and ESI‐TOF HRMS (MICROTOF – Bruker Daltonics).

### 4.2 Synthesis of Compounds 5a–j via Suzuki Cross‐Coupling

For the synthesis of compounds **5a–j**, the brominated intermediates **4a–e** (1.0 mmol), aryl/pyridyl boronic acid (1.2 mmol), Pd(OAc)_2_ (5.0 mol%, 11.2 mg), PPh_3_ (10.0 mol%; 26.2 mg), K_2_CO_3_ (3.0 mmol, 414 mg), and a mixture of 1,4‐dioxane/water/ethanol 5:1:1 (7.0 mL) were combined in a Schlenk flask previously evacuated and back‐filled with nitrogen. The mixture was then stirred at 120°C for 18 h. After cooling to room temperature, the crude reaction was diluted with ethanol and concentrated under vacuum. The crude product was purified by column chromatography on silica gel using hexane/ethyl acetate as mobile phase.

#### 4.2.1 4′‐(imidazo[1,2‐a]pyridin‐2‐Yl)‐[1,1′‐Biphenyl]‐4‐Carbaldehyde (5a)

Yellow solid. Melting point = 160°C (decomp). Yield: 25%. ^1^H NMR (400 MHz, CDCl_3_) δ 9.99 (s, 1H), 8.10 (d, *J* = 6.7 Hz, 1H), 8.02 (d, *J* = 8.4 Hz, 2H), 7.92–7.83 (m, 3H), 7.72 (d, *J* = 8.3 Hz, 3H), 7.65 (d, *J* = 8.4 Hz, 2H), 7.21 (d, *J* = 7.2 Hz, 1H), 6.80 (t, *J* = 6.5 Hz, 1H). ^13^C NMR (101 MHz, CDCl_3_) δ 191.9, 146.6, 145.2, 144.0, 139.4, 135.3, 132.9, 130.3, 127.8, 127.5, 126.7, 125.9, 125.8, 117.2, 113.3, 108.6. HRMS (ESI) *m/z* calculated for C_20_H_15_N_2_O [M + H]^+^: 299.1184; found: 299.1195. FTIR‐ATR (cm^−1^): 3135 (ν C—H), 2804 (ν C—H), 2718 (ν C—H), 1696 (ν C═O), 1599 (ν C═C), 817 (δ C—H).

#### 4.2.2 4′‐(imidazo[1,2‐a]pyridin‐2‐Yl)‐[1,1′‐Biphenyl]‐4‐Carbonitrile (5b)

Yellow solid. Melting point = 201°C (decomp). Yield: 76%. ^1^H NMR (400 MHz, CDCl_3_) δ 8.08 (dt, *J* = 6.8, 1.1 Hz, 1H), 8.03–7.99 (m, 2H), 7.87–7.85 (m, 1H), 7.67–7.59 (m, 7H), 7.18–7.13 (m, 1H), 6.77 (td, *J* = 6.8, 1.0 Hz, 1H). ^13^C NMR (101 MHz, CDCl_3_) δ 145.6, 145.2, 144.6, 138.6, 133.8, 132.6, 127.6, 127.5, 126.7, 125.7, 125.3, 118.9, 117.5, 112.9, 110.9, 108.5. HRMS (ESI) *m/z* calculated for C_20_H_14_N_3_ [M + H]^+^: 296.1188; found: 296.1211. FTIR‐ATR (cm^−1^): 3030 (ν C—H), 2223 (ν C≡N), 1603 (ν C═C), 1476 (ν C═C), 825 (δ C—H).

#### 4.2.3 2‐(4′‐Methoxy‐3′,5′‐Dimethyl‐[1,1′‐Biphenyl]‐4‐Yl)imidazo[1,2‐a]pyridine (5c)

White solid. Melting point = 142°C–145°C. Yield: 63%. ^1^H NMR (400 MHz, CDCl_3_) δ 8.10 (dt, *J* = 6.8, 1.1 Hz, 1H), 8.01–7.97 (m, 2H), 7.87 (s, 1H), 7.66–7.61 (m, 3H), 7.30 (s, 2H), 7.17 (ddd, *J* = 9.0, 6.8, 1.2 Hz, 1H), 6.77 (td, *J* = 6.8, 1.1 Hz, 1H), 3.76 (s, 3H), 2.36 (s, 6H). ^13^C NMR (101 MHz, CDCl_3_) δ 156.7, 145.7, 145.4, 140.45, 136.3, 132.2, 131.2, 127.4, 127.2, 126.4, 125.6, 124.8, 117.5, 112.5, 108.1, 59.8, 16.3. HRMS (ESI) *m/z* calculated for C_22_H_21_N_2_O [M + H]^+^: 329.1654; found: 329.1651. FTIR‐ATR (cm^−1^): 3127 (ν C—H), 2913 (ν C—H), 2821 (ν C—H), 1632 (ν C═C), 1472 (ν C═C), 1369 (δ CH3), 1274 (ν C—O), 1010 (ν C—O), 844 (δ C—H).

#### 4.2.4 2‐(4′‐Methoxy‐[1,1′‐Biphenyl]‐4‐Yl)imidazo[1,2‐a]pyridine (5d)

White solid. Melting point = 198°C (decomp). Yield: 39%. ^1^H NMR (400 MHz, CDCl_3_) δ 8.11 (d, *J* = 6.8 Hz, 1H), 8.02–7.98 (m, 2H), 7.87 (s, 1H), 7.64 (dd, *J* = 8.4, 6.4 Hz, 3H), 7.60–7.56 (m, 2H), 7.19–7.12 (m, 1H), 7.01 – 6.95 (m, 2H), 6.76 (td, *J* = 6.8, 1.0 Hz, 1H), 3.85 (s, 3H). ^13^C NMR (101 MHz, CDCl_3_) δ 159.2, 145.7, 145.4, 140.4, 133.3, 131.9, 128.0, 126.9, 126.4, 125.6, 124.8, 117.5, 114.3, 112.5, 108.1, 55.4. HRMS (ESI) *m/z* calculated for C_20_H_17_N_2_O [M + H]^+^: 301.1341; found: 301.1360. FTIR‐ATR (cm^−1^): 3135 (ν C—H), 2873 (ν C—H), 1603 (ν C═C), 1478 (ν C═C), 1371 (δ CH3), 1248 (ν C—O), 10 345 (ν C—O), 827 (δ C—H).

#### 4.2.5 2‐(4‐(pyridin‐4‐Yl)phenyl)imidazo[1,2‐a]pyridine (5e)

White solid. Melting point = 210°C (decomp). Yield: 11%. ^1^H NMR (400 MHz, CDCl_3_) δ 8.66 (dd, *J* = 4.6, 1.6 Hz, 2H), 8.14 (d, *J* = 6.8 Hz, 1H), 8.08 (d, *J* = 8.4 Hz, 2H), 7.93 (s, 1H), 7.73 (d, *J* = 8.4 Hz, 2H), 7.65 (d, *J* = 9.1 Hz, 1H), 7.56 (dd, *J* = 4.6, 1.6 Hz, 2H), 7.23–7.16 (m, 1H), 6.81 (td, *J* = 6.8, 0.9 Hz, 1H). ^13^C NMR (101 MHz, CDCl_3_) δ 145.3, 144.1, 135.4, 131.1, 130.3, 129.0, 126.5, 124.5, 123.4, 123.0, 120.9, 118.0, 108.8. HRMS (ESI) *m/z* calculated for C_18_H_14_N_3_ [M + H]^+^: 272.1188; found: 272.1200. FTIR‐ATR (cm^−1^): 3130 (ν C—H), 3034 (ν C—H), 1589 (ν C═C), 1475 (ν C═C), 812 (δ C—H).

#### 4.2.6 2‐(4‐(pyridin‐3‐Yl)phenyl)imidazo[1,2‐a]pyridine (5f)

Yellow solid. Melting point = 188°C (decomp). Yield: 19%. ^1^H NMR (400 MHz, CDCl_3_) δ 8.90 (d, *J* = 1.8 Hz, 1H), 8.58 (dd, *J* = 4.8, 1.4 Hz, 1H), 8.12 (d, *J* = 6.8 Hz, 1H), 8.06 (d, *J* = 8.2 Hz, 2H), 7.94 – 7.89 (m, 2H), 7.65 (dd, *J* = 8.2, 6.8 Hz, 3H), 7.36 (dd, *J* = 7.9, 4.8 Hz, 1H), 7.21–7.15 (m, 1H), 6.78 (td, *J* = 6.8, 0.9 Hz, 1H). ^13^C NMR (101 MHz, CDCl_3_) δ 147.4, 147.1, 144.8, 144.0, 136.2, 135.2, 133.2, 132.6, 126.4, 125.7, 124.6, 123.9, 122.6, 116.6, 111.6, 107.4. HRMS (ESI) *m/z* calculated for C_18_H_14_N_3_ [M + H]^+^: 272.1188; found 272.1199. FTIR‐ATR (cm^−1^): 3128 (ν C—H), 3055 (ν C—H), 1632 (ν C═C), 1472 (ν C═C), 1273 (ν C—N), 802 (δ C—H).

#### 4.2.7 6‐Methyl‐2‐(4‐(pyridin‐4‐Yl)phenyl)imidazo[1,2‐a]pyridine (5g)

White solid. Melting point = 131°C–134°C. Yield: 15%. ^1^H NMR (400 MHz, CDCl_3_) δ 8.67 (d, *J* = 5.9 Hz, 1H), 8.06 (d, *J* = 8.3 Hz, 1H), 7.93 (s, 1H), 7.85 (s, 1H), 7.73 (d, *J* = 8.3 Hz, 1H), 7.60–7.54 (m, 1H), 7.05 (d, *J* = 9.3 Hz, 1H), 2.34 (s, 1H). ^13^C NMR (101 MHz, CDCl_3_) δ 150.2, 148.0, 144.9, 144.6, 137.2, 134.8, 128.2, 127.3, 126.6, 123.3, 122.3, 121.4, 116.9, 108.3, 18.1. HRMS (ESI) *m/z*, calculated for C_19_H_16_N_3_ [M + H]^+^: 286.1344; found 286.1343. FTIR‐ATR (cm^−1^): 3125 (ν C—H), 3080 (ν C—H), 2922 (ν C—H), 1591 (ν C═C), 1404 (ν C═C), 1209 (ν C—C), 810 (δ C—H).

#### 4.2.8 6‐Chloro‐2‐(4‐(pyridin‐4‐Yl)phenyl)imidazo[1,2‐a]pyridine (5h)

White solid. Melting point = 223°C (decomp). Yield: 25%. ^1^H NMR (400 MHz, CDCl_3_) δ 8.67 (dd, *J* = 4.5, 1.7 Hz, 2H), 8.18 (d, *J* = 2.0 Hz, 1H), 8.06 (d, *J* = 8.5 Hz, 2H), 7.88 (s, 1H), 7.73 (d, *J* = 8.5 Hz, 2H), 7.59 (d, *J* = 9.6 Hz, 1H), 7.55 (dd, *J* = 4.5, 1.7 Hz, 2H), 7.16 (dd, *J* = 9.6, 2.0 Hz, 1H). ^13^C NMR (101 MHz, CDCl_3_) δ 150.3, 147.8, 146.0, 144.2, 137.7, 134.1, 127.4, 126.7, 126.4, 123.4, 121.4, 120.8, 118.0, 108.9. HRMS (ESI) *m/z* calculated for C_18_H_13_ClN_3_ [M + H]^+^: 306.0798; found 306.0807. FTIR‐ATR (cm^−1^): 3132 (ν C—H), 3078 (ν C—H), 3034 (ν C—H), 1591 (ν C═C), 1474 (ν C═C), 810 (δ C—H), 800 (ν C—Cl).

#### 4.2.9 2‐(3‐(pyridin‐4‐Yl)phenyl)imidazo[1,2‐a]pyridine (5i)

White solid. Melting point = 154°C–156°C. Yield: 40%. ^1^H NMR (400 MHz, CDCl_3_) δ 8.66 (dd, *J* = 4.5, 1.7 Hz, 2H), 8.26 (t, *J* = 1.6 Hz, 1H), 8.11 (dt, *J* = 6.8, 1.1 Hz, 1H), 7.97 (dt, *J* = 7.6, 1.6 Hz, 1H), 7.91 (s, 1H), 7.64 (dd, *J* = 9.1, 1.1 Hz, 1H), 7.60 – 7.56 (m, 3H), 7.52 (t, *J* = 7.6 Hz, 1H), 7.18 (ddd, *J* = 9.1, 6.8, 1.1 Hz, 1H), 6.78 (td, *J* = 6.8, 1.1 Hz, 1H). ^13^C NMR (101 MHz, CDCl_3_) δ 150.2, 148.2, 145.8, 145.2, 138.7, 134.8, 129.5, 126.6, 126.5, 125.7, 125.0, 124.7, 121.8, 117.6, 112.6, 108.5. HRMS (ESI) *m/z* calculated for C_18_H_14_N_3_ [M + H]^+^: 272.1188; found 272.1203. FTIR‐ATR (cm^−1^): 3069 (ν C—H), 3030 (ν C—H), 1593 (ν C═C), 1462 (ν C═C), 825 (δ C—H), 783 (δ C—H), 750 (δ C—H).

#### 4.2.10 6‐Chloro‐2‐(3‐(pyridin‐4‐Yl)phenyl)imidazo[1,2‐a]pyridine (5j)

Brown solid. Melting point = 205°C (decomp). Yield: 24%. ^1^H NMR (400 MHz, CDCl_3_) δ 8.67 (dd, *J* = 4.5, 1.6 Hz, 2H), 8.24 (t, *J* = 1.6 Hz, 1H), 8.17 (dd, *J* = 2.0, 0.8 Hz, 1H), 7.95 (dt, *J* = 7.7, 1.6, 1.4 Hz, 1H), 7.89 (s, 1H), 7.61–7.52 (m, 5H), 7.15 (dd, *J* = 9.6, 2.0 Hz, 1H). ^13^C NMR (101 MHz, CDCl_3_) δ 150.2, 148.2, 146.2, 144.1 138.8, 134.3, 129.6, 126.8, 126.6, 126.4, 124.7, 123.4, 121.8, 120.8, 117.9, 108.8. HRMS (ESI) *m/z* calculated for C_18_H_13_ClN_3_ [M + H]^+^: 306.0798; found 306.0815. FTIR‐ATR (cm^−1^): 3140 (ν C—H), 3057 (ν C—H), 1597 (ν C═C), 1398 (ν C═C), 1068 (ν C—N), 825 (δ C—H), 795 (ν C—Cl), 781 (δ C—H).

### 4.3 In Vitro Anti‐*T. cruzi* Activity

#### 4.3.1 Compounds Preparation for Biological Tests

Stock solutions of compounds **5a–j** were prepared in dimethylsulfoxide (DMSO) for the in vitro assays against *T. cruzi*, certifying the final solvent concentration remained below 0.6% to avoid toxicity to mammalian cell and parasites [38]. Bz was used as reference drug, and aliquots were stored at –20°C.

#### 4.3.2 Mammalian Cell Cultures

To assess compound cytotoxicity against host mammalian cells and the antiparasitic activity of the studied compounds against intracellular forms, L929 mouse fibroblast monolayers were cultured (4 × 103 cells/well) in 96‐well microplates. The cells were maintained at 37°C in phenol red‐free RPMI‐1640 medium (pH 7.2–7.4) supplemented with 10% fetal bovine serum and 2 mM glutamine (RPMIS), following previously established protocols [38, 39]. The phenotypic studies were strictly conducted according to the FIOCRUZ Committee of Ethics for the Use of Animals guidelines (CEUA L038‐2017).

#### 4.3.3 Parasites and Infection of the Cell Cultures

Trypomastigote forms of *Trypanosoma cruzi* (Y strain ‐ Discrete Typing Unit – DTU II) were obtained by cardiac puncture of infected mice at the parasitemia peak [40]. Tissue culture‐derived trypomastigotes (Tulahuen strain expressing the *E. coli* β‐galactosidase gene, DTU VI) were maintained in L929 cells and harvested from the supernatant 96 h postinfection, as previously described [38]. Briefly, L929 cells were seeded at 4 × 103 cells/well and, after 24 h, were infected with trypomastigotes at a 10:1 ratio. Following an initial 24‐h incubation at 37°C, the monolayers were washed to remove noninternalized parasites. The cultures were then maintained at 37°C until the newly released parasites were collected from the supernatant.

#### 4.3.4 In Vitro Cytotoxicity Tests

L929 cell cultures were incubated for 96 h at 37°C with different compound concentrations (up to 200 µM) diluted in phenol red‐free Dulbecco's Modified Eagle Medium (DMEM). Following incubation, cell morphology was evaluated by light microscopy, and viability was determined using the AlamarBlue assay. Briefly, 10 μL of AlamarBlue was added to each well, followed by an additional 24‐h incubation period, after which absorbance was measured at 570 and 600 nm. Negative controls, consisting of DMEM alone or containing the highest compound concentration in the absence of cells, were included to monitor potential interference. Results were expressed as the percentage difference between treated and vehicle‐control cells, following the manufacturer's instructions. The LC_50_ value was defined as the concentration required to reduce cellular viability by 50%. All assays were performed at least twice in triplicate [41].

#### 4.3.5 Anti‐*T. cruzi* Activity Analysis

To evaluate activity against intracellular forms, L929 cell cultures were infected with trypomastigotes at a 10:1 ratio for 2 h, after which noninternalized parasites were removed by replacing the RPMI medium. Following a 48‐h incubation period, the infected cultures were treated with compounds. Initially, a fixed‐concentration screening was performed at 10 µM and compounds that reduced the parasite load by 30% were subsequently evaluated in dose–response curves (up to 10 µM, using 1:2 serial dilutions). The cultures were maintained for 96 h at 37°C under a 5% CO_2_ atmosphere. Bz and DMSO were used as positive and negative controls, respectively. To determine parasite viability, 50 µL of chlorophenol red‐β‐D‐galactopyranoside (CPRG) was added to each well, and absorbance was measured at 570 nm. Activity was expressed as EC_50_ values, representing the concentration required to reduce parasite viability by 50% [38, 39]. All assays were performed in triplicate and repeated in at least two independent experiments.

#### 4.3.6 Data Analysis and EC_50_ Calculation

Nonlinear regression analysis was performed using Prism GraphPad software to calculate EC_50_ values and 95% confidence intervals. All calculations were based on data derived from at least two independent experiments performed in triplicate.

### 4.4 Cruzain Inhibition Assay

Cruzain, a recombinant form of protein cruzipain (EC 3.4.22.51,) was expressed and purified according to previous procedures [42, 43] in the Interdisciplinary Center for Biochemical Research of University of Mogi das Cruzes. The substrato Z‐Phe‐Arg 7‐amido‐4‐methylcoumarin hydrochloride (Z‐Phe‐Arg‐AMC) was acquired from TargetMol (USA).

For the screening assay, cruzain was preincubated for 5 min at 35°C in 100 mM sodium acetate buffer (pH = 5.5) containing 2.5 mM dithiothreitol (DTT). Enzymatic activity was monitored via the hydrolysis of the fluorogenic substrate Z‐FR‐AMC (*λ*_ex_ = 360 nm and *λ*_em_ = 480 nm) using a Hitachi F2500 spectrofluorometer. Initially, the compounds were tested at fixed concentrations of 10 and 100 µM. For the determination of inhibitory potential (IC_50_), the enzyme was treated with increasing inhibitor concentrations until the decay in enzymatic activity stabilized. IC_50_ values were determined by applying increasing concentrations of the tested compounds. The data points from the concentration–response curves of the experiments were analyzed by nonlinear regression using GraFit software, according to Equation (1). The reported IC_50_ values are expressed as mean ± standard deviation (SD) derived from the fitted curves [43].

(1) $$y=\frac{100\%}{1+{\left(\frac{x}{{\text{IC}}_{50}}\right)}^{S}}$$

### 4.5 Molecular Docking Studies

The structures of compounds **5a–j** were obtained using the MarvinSketch program and the protonation states on pH = 5.5 (pH of the enzyme inhibition assay) were verified. Then, the molecules were generated using the Discovery Studio Visualizer program and minimized using the semiempirical PM7 method in the MOPAC package through Mercury.

The crystal structure of cruzain with a resolution of 1.2 Å and cocrystallized with [1‐(1‐benzyl‐3‐hydroxy‐2‐oxo‐propylcarbamoyl)‐2‐phenyl‐ethyl]‐carbamic acid benzyl ester was obtained from Protein Data Bank (PDB ID: 1ME4) [44]. The Ramachandran plot analysis (Figure S48) was used to verify the quality of the protein structure. The computational method was validated by redocking the cocrystallized ligand, using a semiflexible protocol in GOLD software. The GoldScore scoring function was chosen to proceed because it provided the lowest RMSD (0.9574 Å) and showed better reproduction of the interactions. Lastly, the molecular docking of the compounds was performed, using the position of the cocrystallized ligand as the center of the interaction sphere and the size of each ligand as the radius. Finally, the conformation with the highest fitness values among the 100 possible poses was chosen for analysis.

## Funding

This study was supported by Fundação Carlos Chagas Filho de Amparo à Pesquisa do Estado do Rio de Janeiro (SEI‐260003/003400/2022 and SEI‐260003/001526/2022), Coordenação de Aperfeiçoamento de Pessoal de Nível Superior (001), and Fundação de Amparo à Pesquisa do Estado de São Paulo (2014/02205‐1, 2016/25112‐4, and 2021/01503‐2).

## Conflicts of Interest

The authors declare no conflicts of interest.

## Supporting information

Supplementary Material

## Data Availability

The data that supports the findings of this study are available in the Supporting Information of this article.
