# Supplementary material for: Pyridyl‐Imidazopyridine Derivatives Displaying Submicromolar Activity Against Trypanosoma cruzi
Source: ChemMedChem. 2026 Jul 10;21(13):e70377. doi: 10.1002/cmdc.70377 (PMC13354723; doi:10.1002/cmdc.70377)
Supplement: Supplementary file 1 — Supplementary Material [file CMDC-21-e70377-s001.pdf]

Supporting Information
Pyridyl-Imidazopyridine Derivatives Displaying Submicromolar Activity against Trypanosoma cruzi

Ana Carolina Rocha Barreto[1], Kelly Lopes Figueira[1], Raquel Azevedo[2], Ketlym da Conceição[2], Ludmila de Ferreira de Almeida Fiuza[2], Luan dos Santos Vianna[3], Thiago Apolinário de Moraes[3], Wagner Alves de Souza Júdice[3], Maria de Nazaré Correia Soeiro[2], and Jones Limberger[1,*]

[1]Department of Chemistry, Pontifical Catholic University of Rio de Janeiro, Rua Marquês de São Vicente, 225, Gávea, 22451-900, Rio de Janeiro-RJ, Brazil

[2]Cell Biology Laboratory, Oswaldo Cruz Institute, Oswaldo Cruz Foundation, Avenida Brasil, 4.365, Manguinhos, 21040-900, Rio de Janeiro-RJ, Brazil

[3]Interdisciplinary Center for Biochemical Research, University of Mogi das Cruzes, Av Dr. Cândido Xavier de Almeida e Souza, 200, 08780-991, Mogi das Cruzes, Brazil

---

^1H and ^{13}C NMR Spectra 2

High-Resolution Mass Spectra 12

FTIR-ATR Spectra 17

Dose-response curves 22

Activity against cruzain 23

Ramachandran plot for 1ME4 26

Goldscores 27

Key amino acid residues involved in the binding 28

^{1}H and ^{13}C NMR Spectra

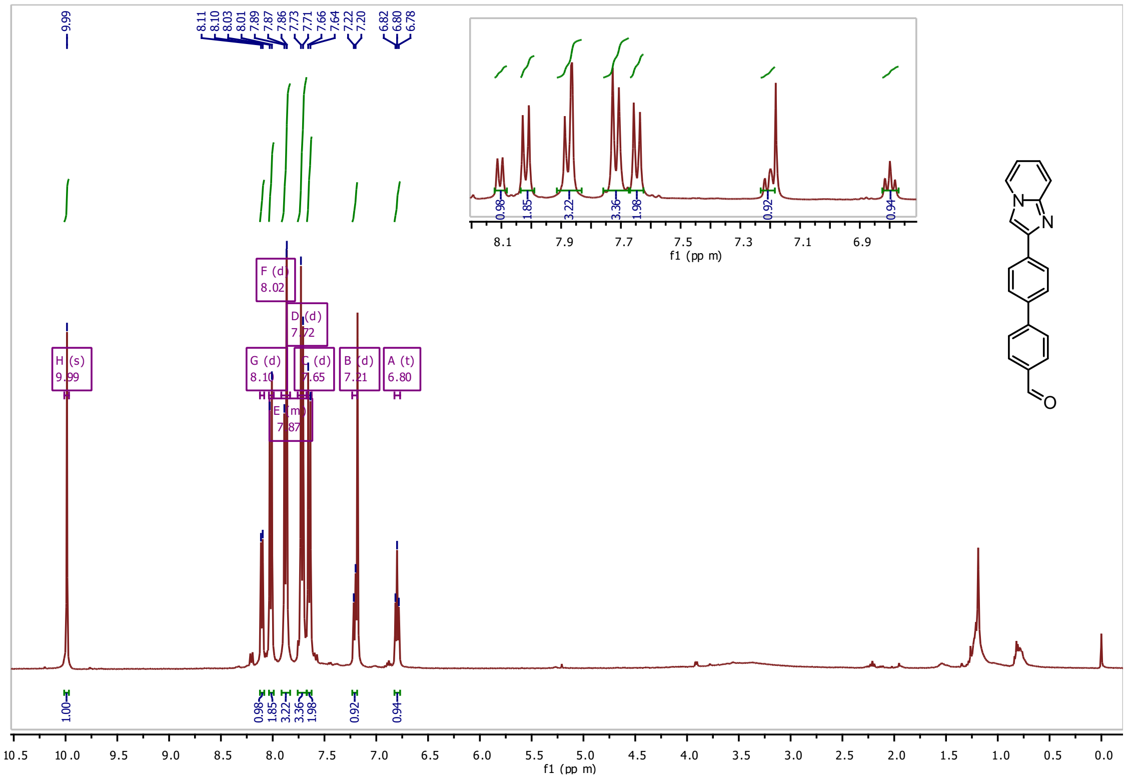

Figure S1 - ^{1}H NMR (400 MHz, $CDCl_3$) of compound **5a**

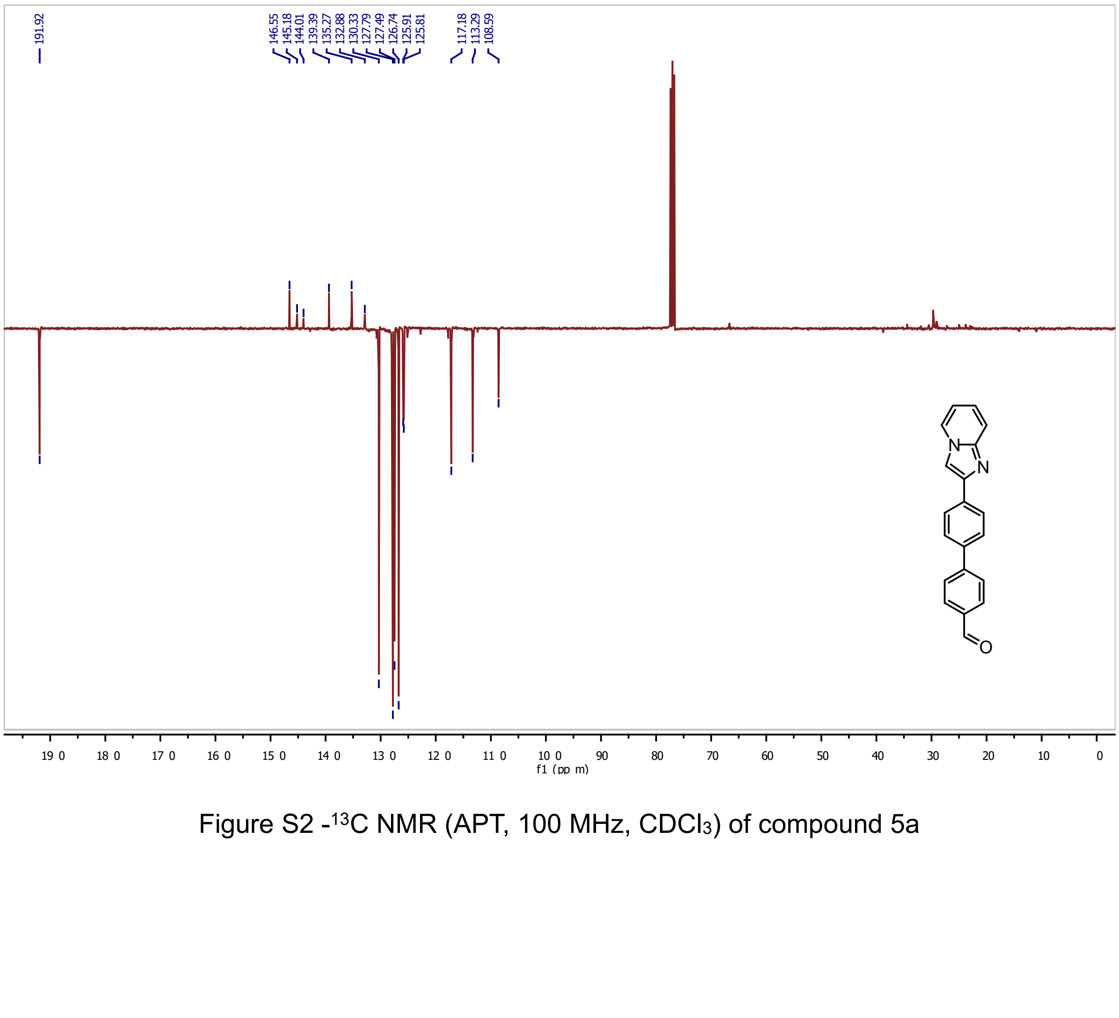

Figure S2 -^{13}C NMR (APT, 100 MHz, $CDCl_3$) of compound 5a

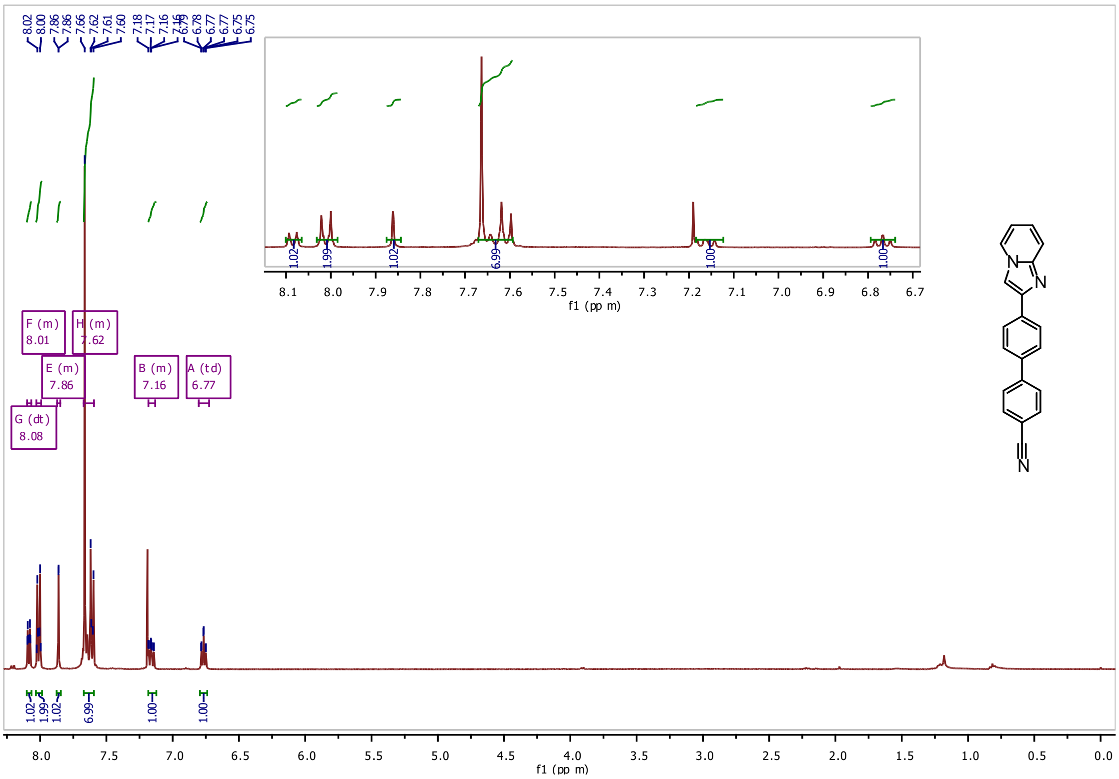

Figure S3 - ^{1}H NMR (400 MHz, $CDCl_3$) of compound **5b**

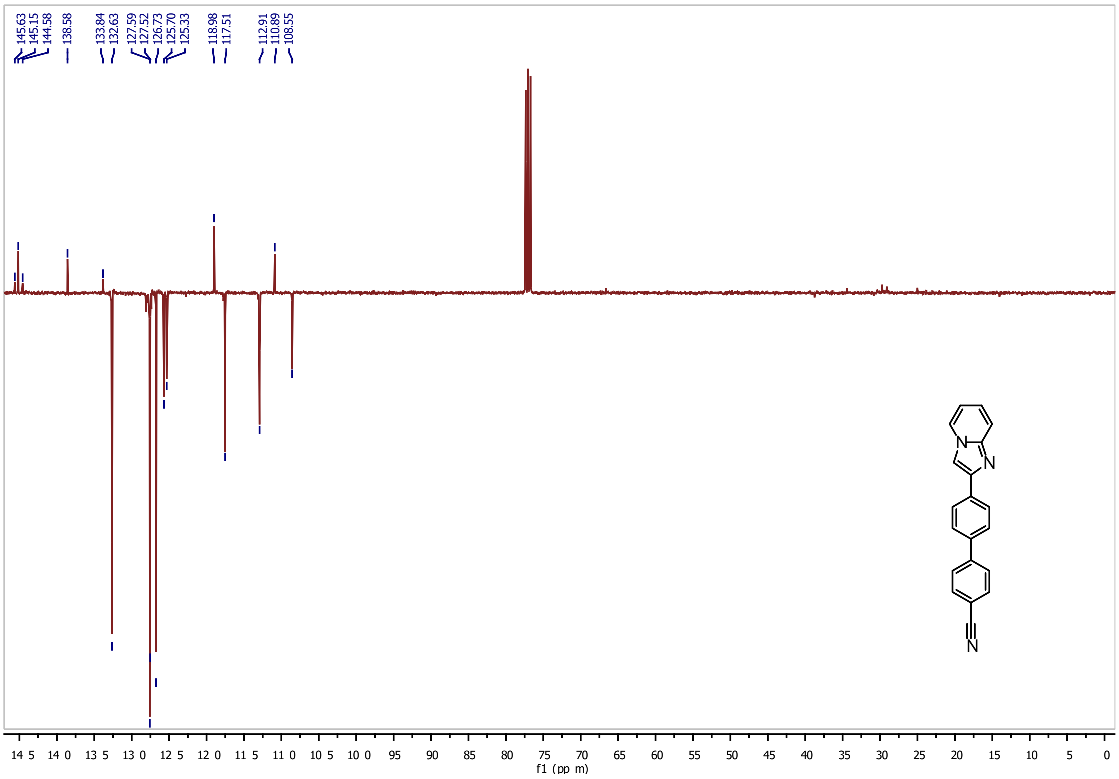

Figure S4 - ^{13}C NMR (APT, 100 MHz, $CDCl_3$) of compound **5b**

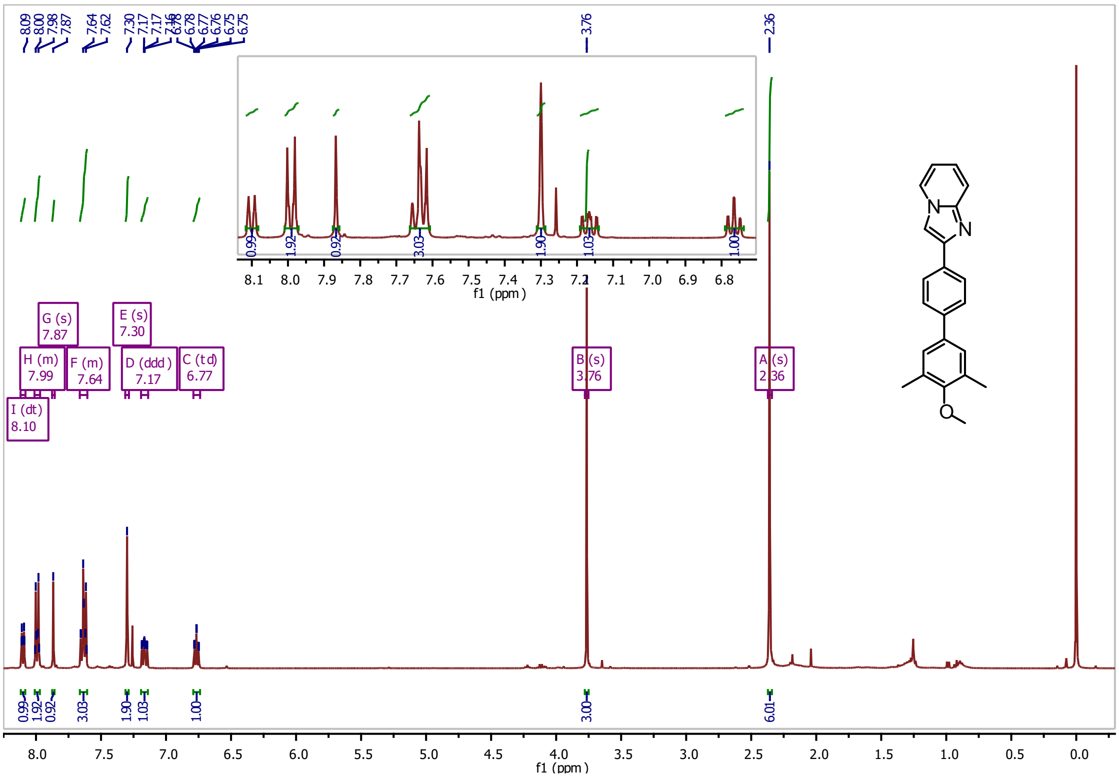

Figure S5 - ^{1}H NMR (400 MHz, $CDCl_3$) of compound **5c**

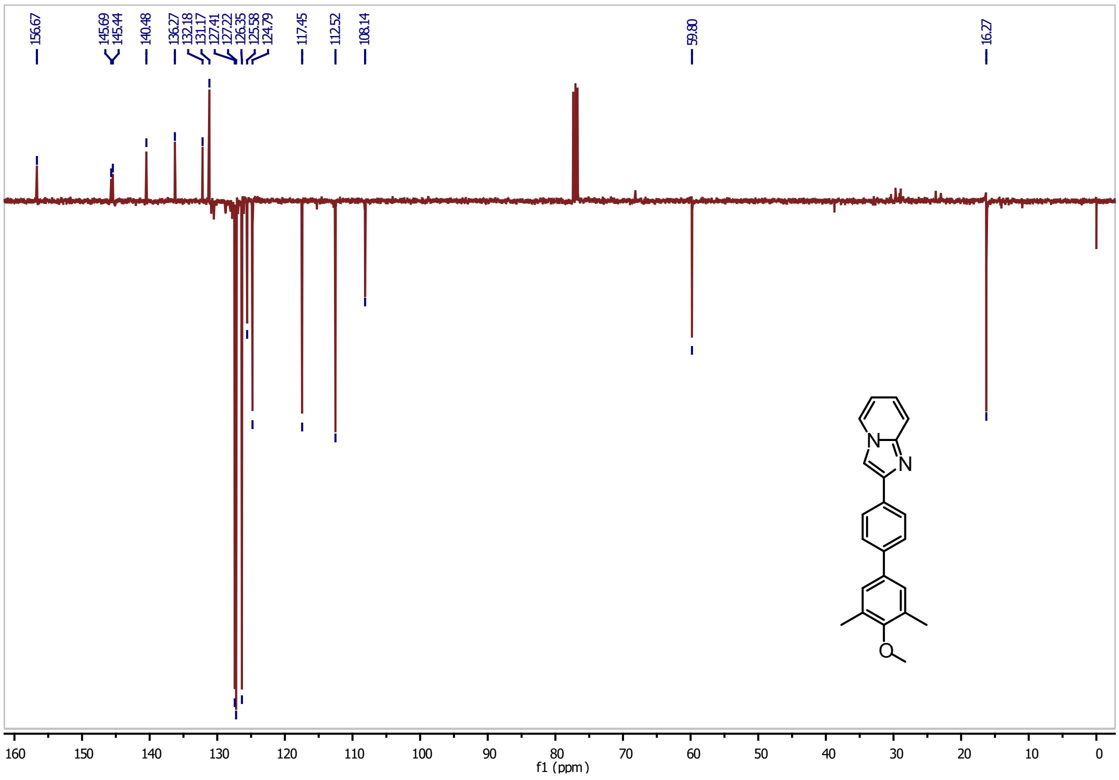

Figure S6 - ^{13}C NMR (APT, 100 MHz, $CDCl_3$) of compound **5c**

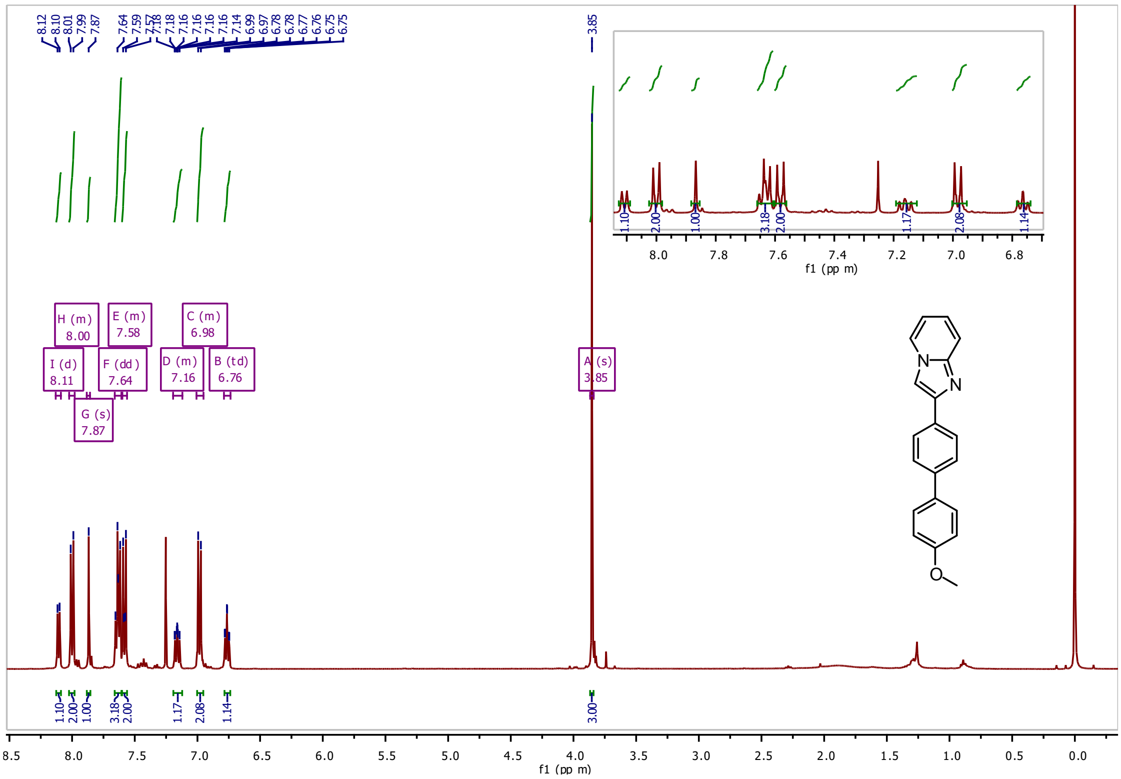

Figure S7 - ^{1}H NMR (400 MHz, $CDCl_3$) of compound **5d**

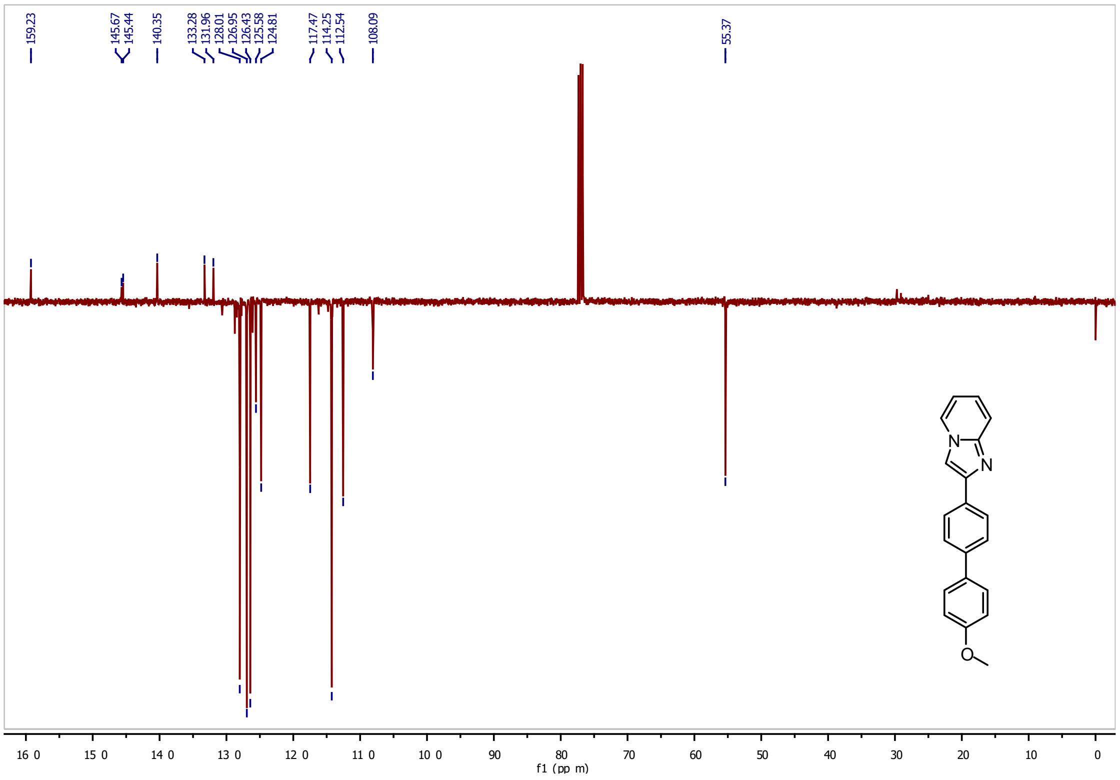

Figure S8 - ^{13}C NMR (APT, 100 MHz, $CDCl_3$) of compound **5d**

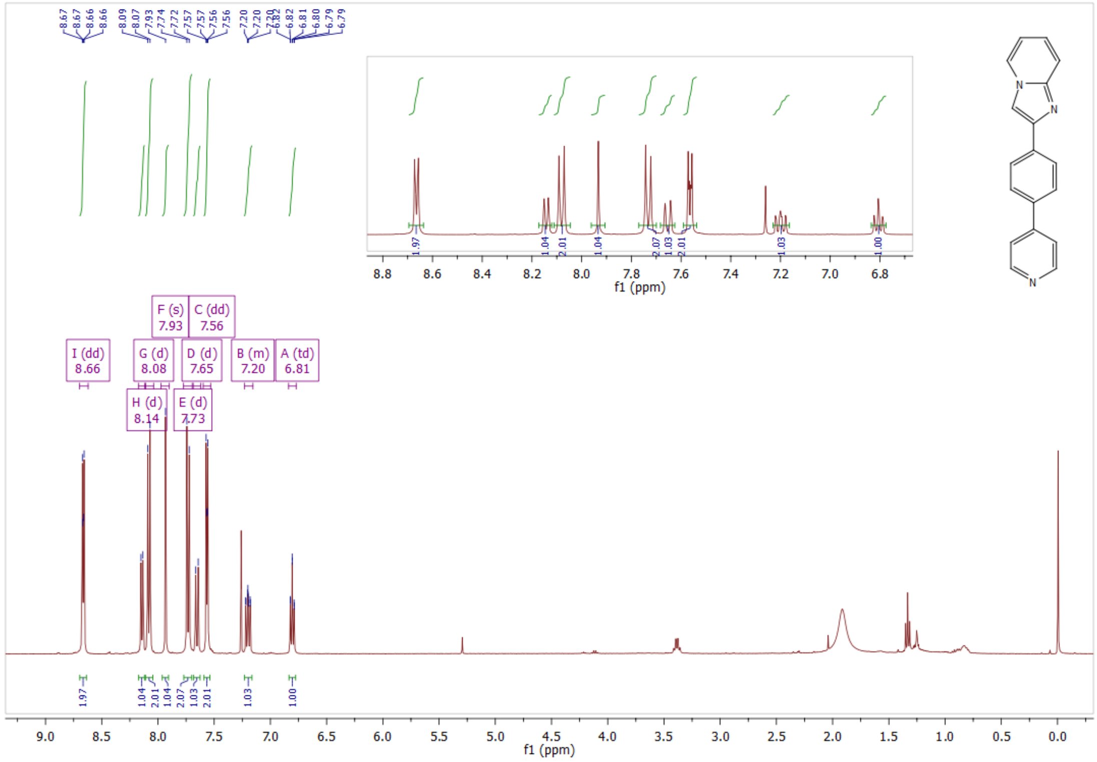

Figure S9 - ^{1}H NMR (400 MHz, $CDCl_3$) of compound **5e**

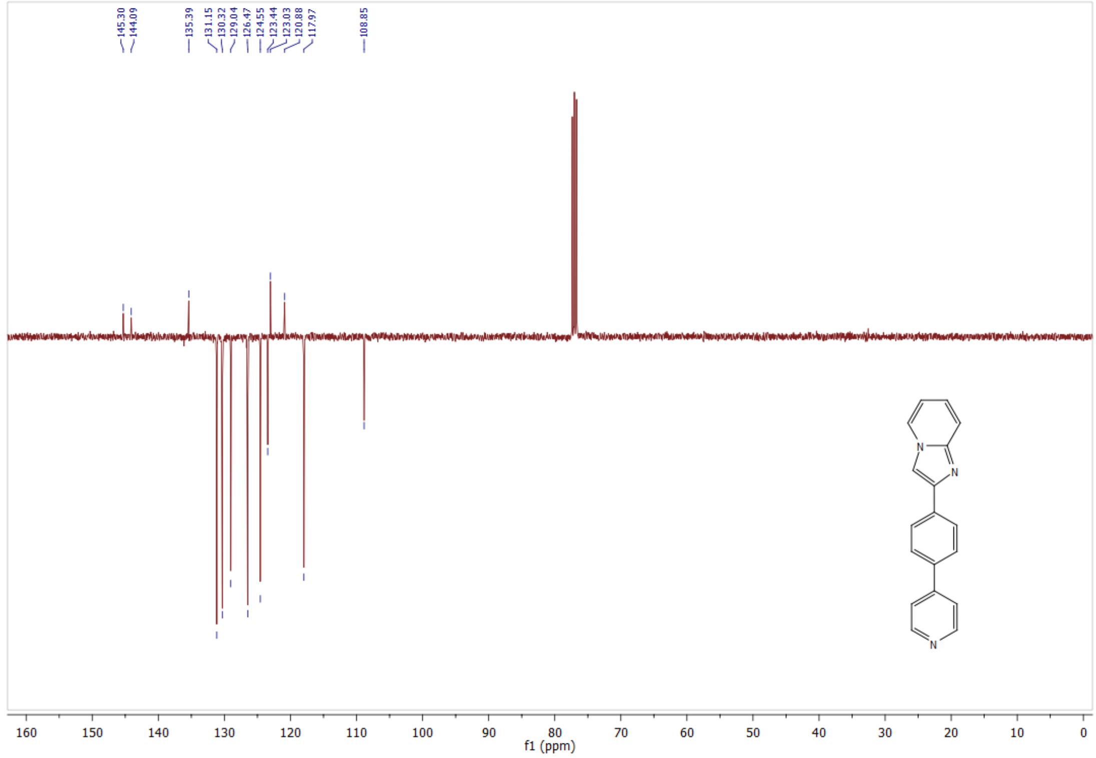

Figure S10 - ^{13}C NMR (APT, 100 MHz, $CDCl_3$) of compound **5e**

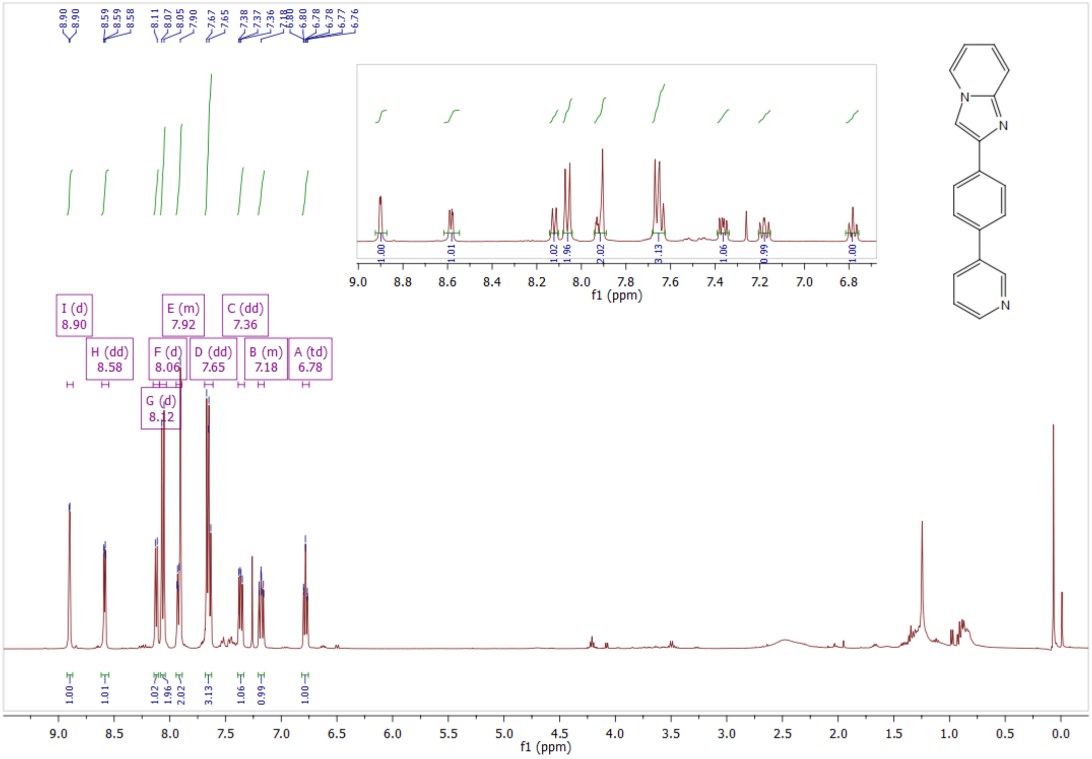

Figure S11 - ^{1}H NMR (400 MHz, $CDCl_3$) of compound **5f**

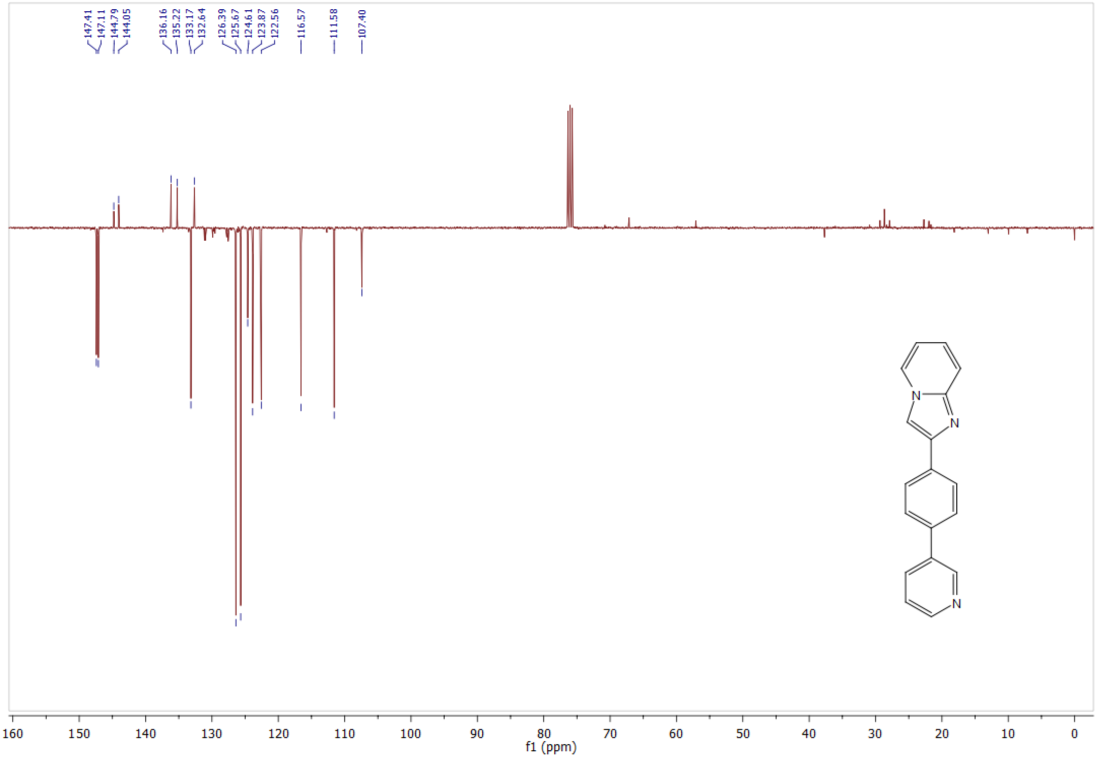

Figure S12 - ^{13}C NMR (APT, 100 MHz, $CDCl_3$) of compound **5f**

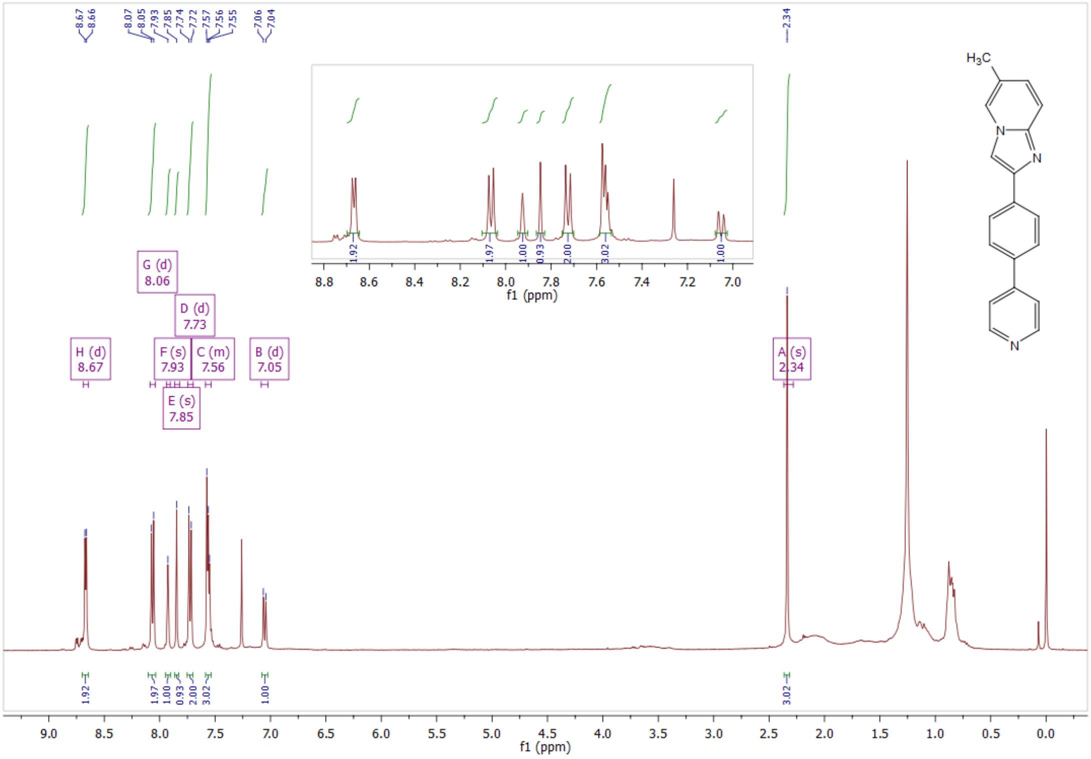

Figure S13 - ^{1}H NMR (400 MHz, $CDCl_3$) of compound **5g**

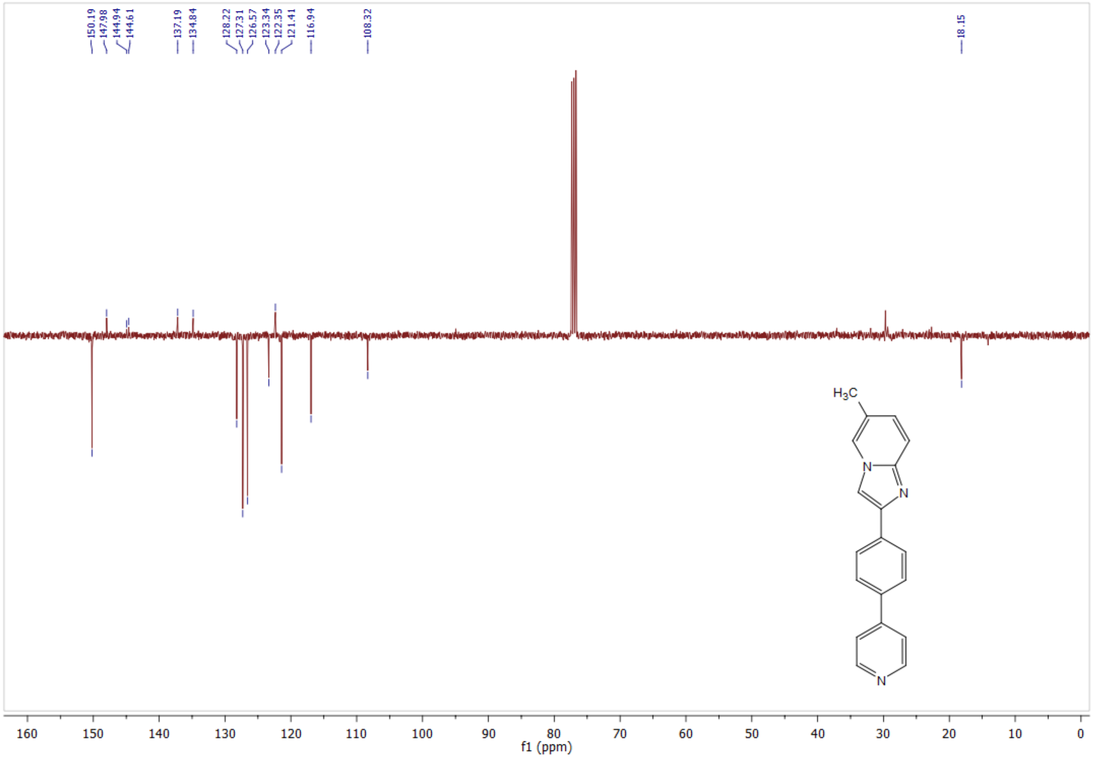

Figure S14 - ^{13}C NMR (APT, 100 MHz, $CDCl_3$) of compound **5g**

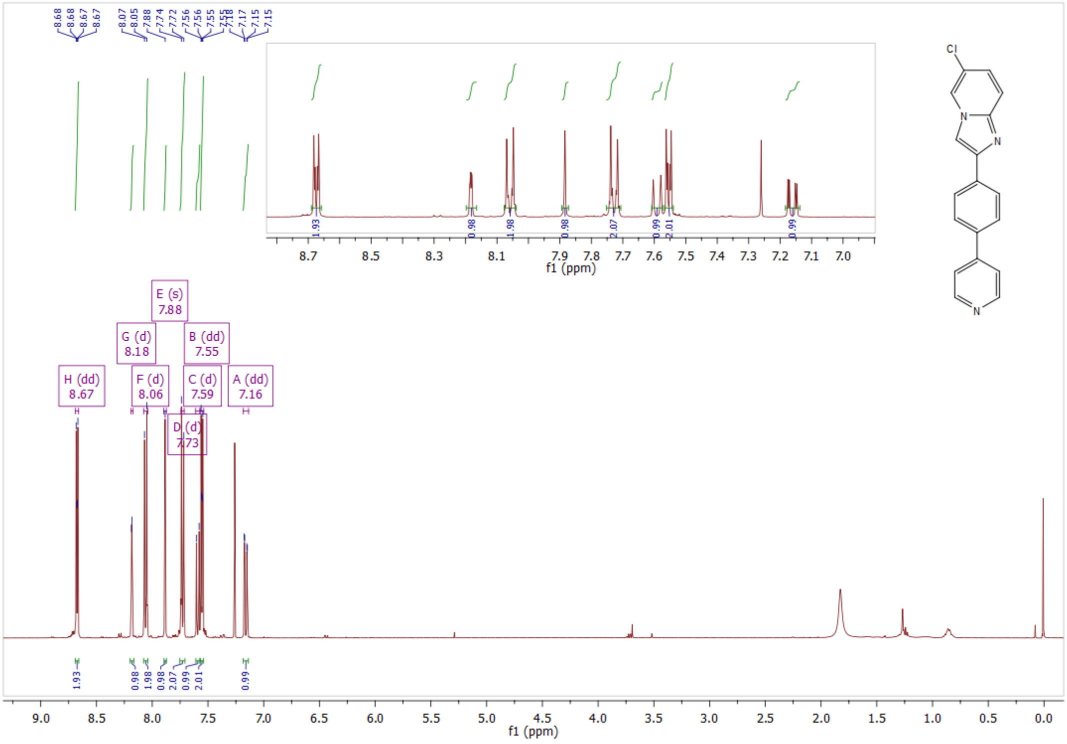

Figure S15 - ^{1}H NMR (400 MHz, $CDCl_3$) of compound **5h**

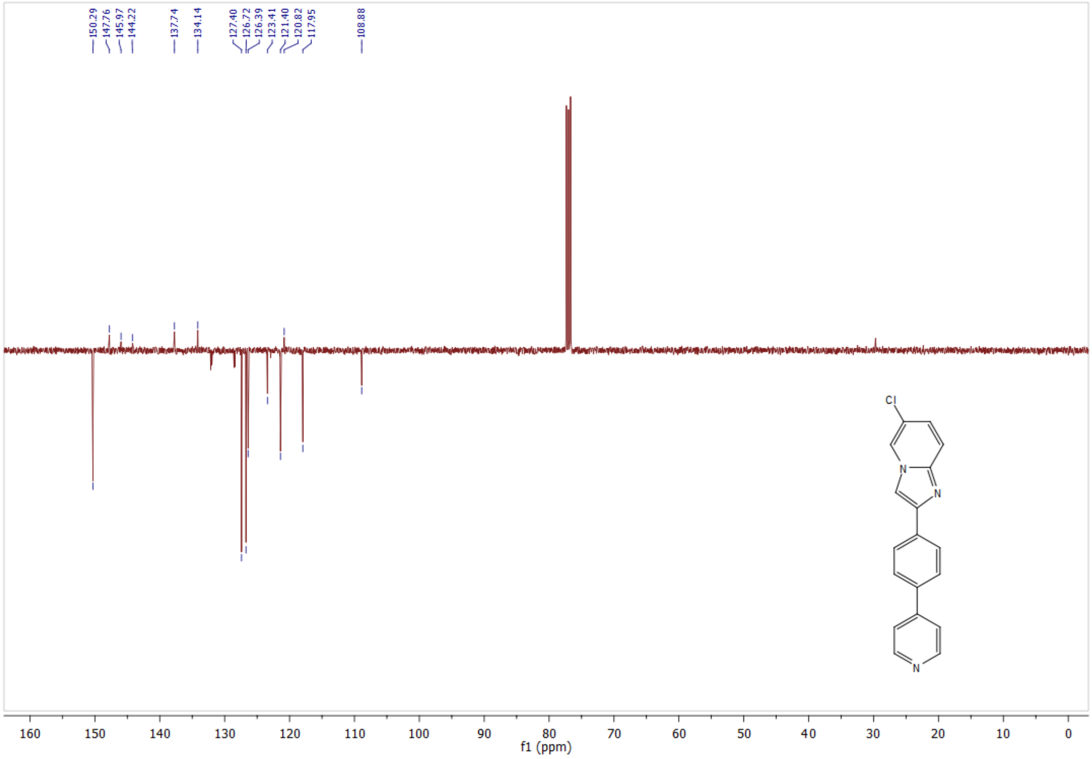

Figure S16 - ^{13}C NMR (APT, 100 MHz, $CDCl_3$) of compound **5h**

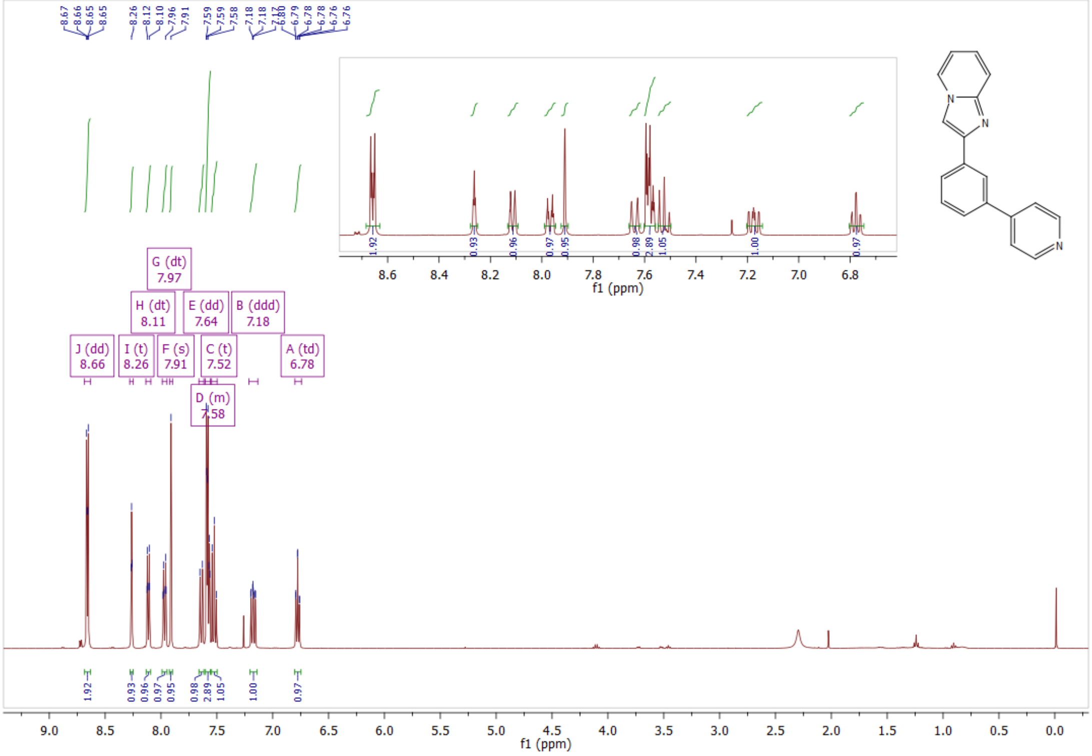

Figure S17 - ^{1}H NMR (400 MHz, $CDCl_3$) of compound **5i**

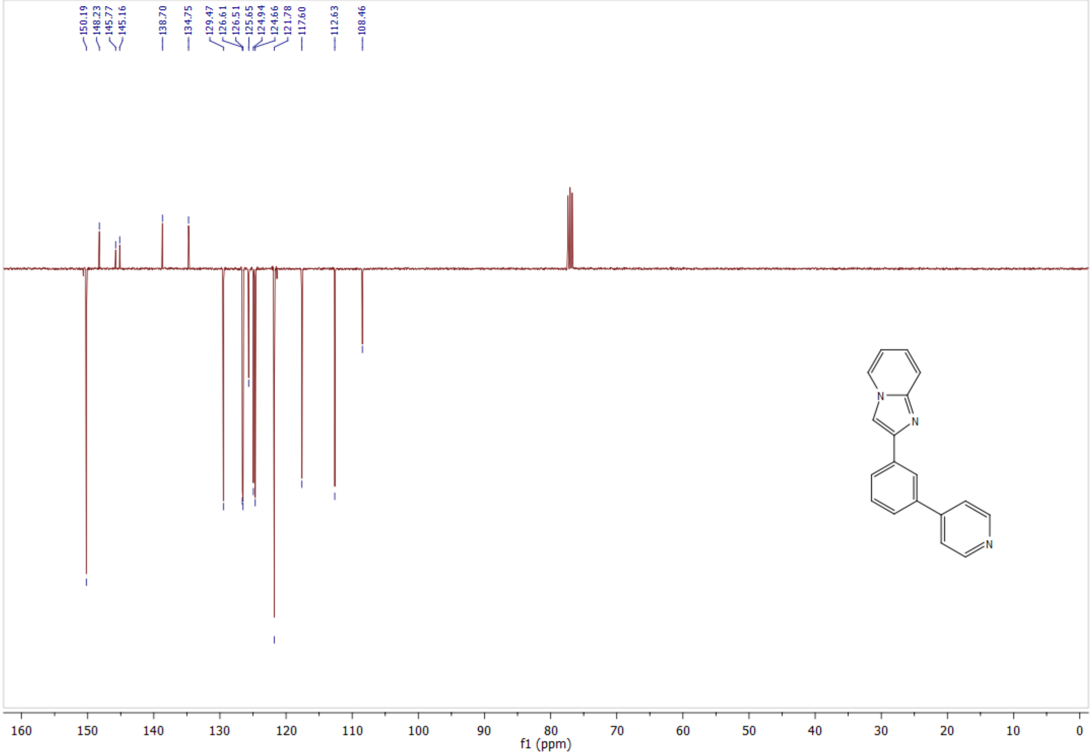

Figure S18 - ^{13}C NMR (APT, 100 MHz, $CDCl_3$) of compound **5i**

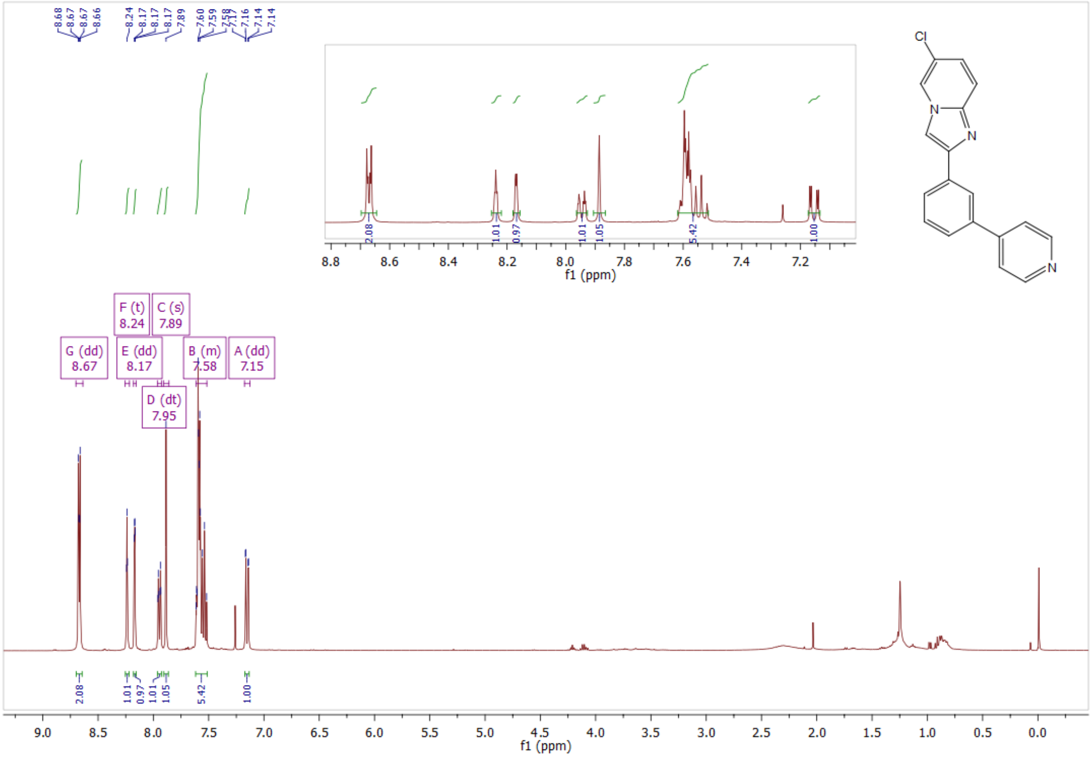

Figure S19 - ^{1}H NMR (400 MHz, $CDCl_3$) of compound **5j**

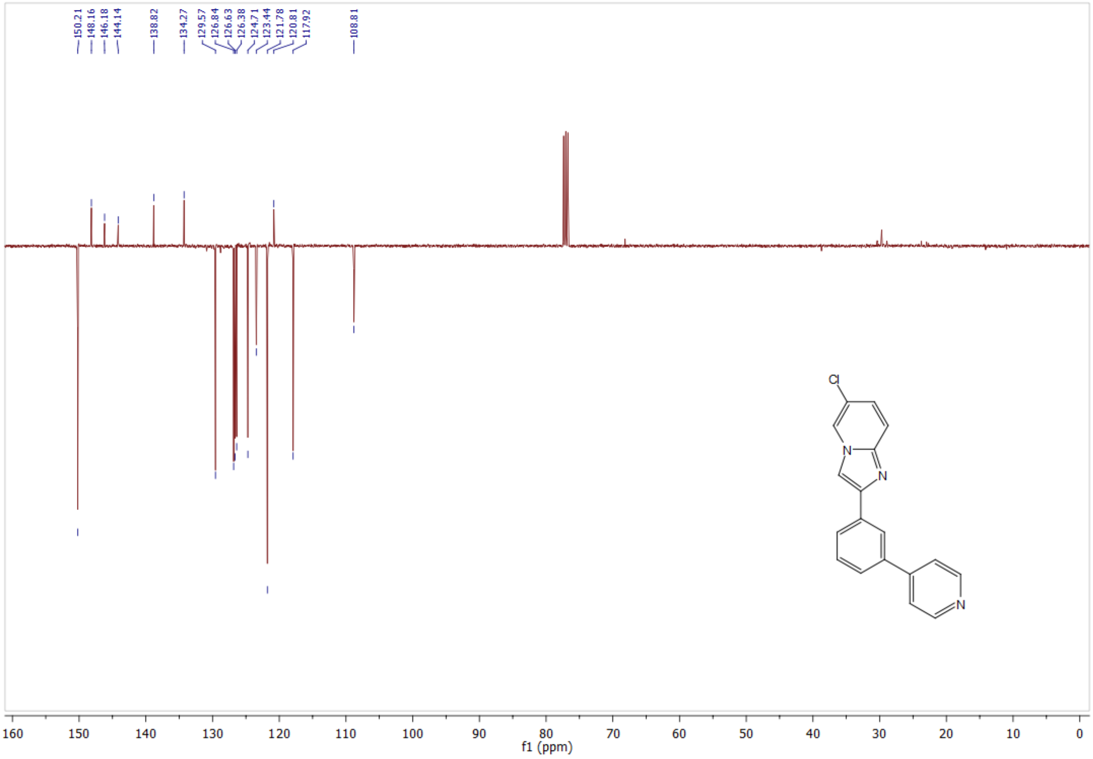

Figure S20 - ^{13}C NMR (APT, 100 MHz, $CDCl_3$) of compound **5j**

High-Resolution Mass Spectra

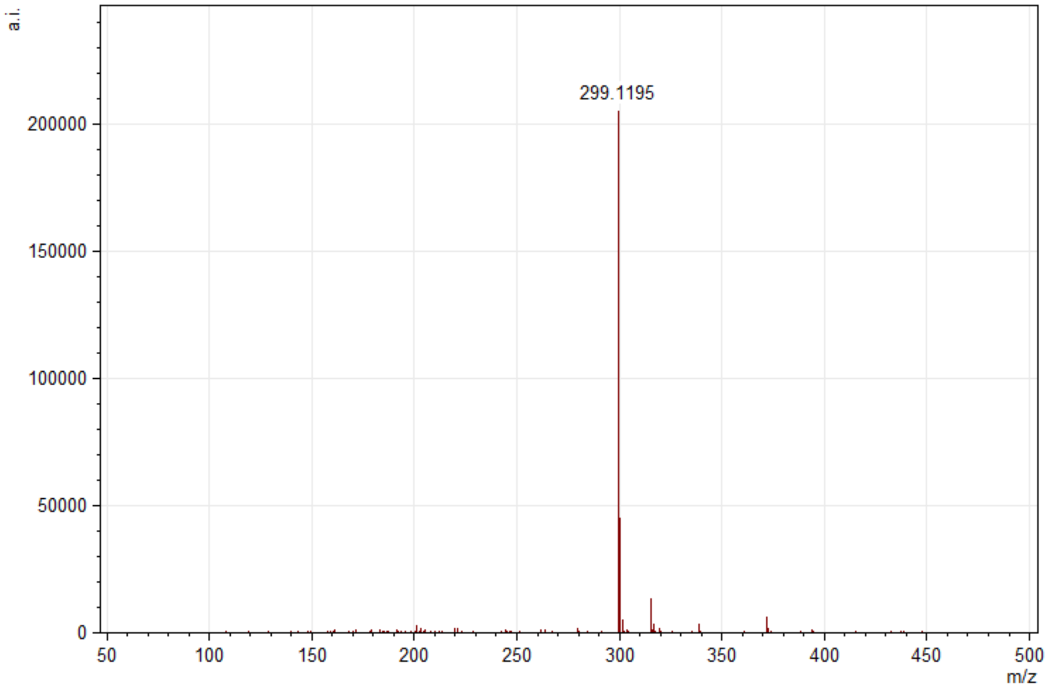

Figure S21 - HRMS (ESI) of compound **5a**

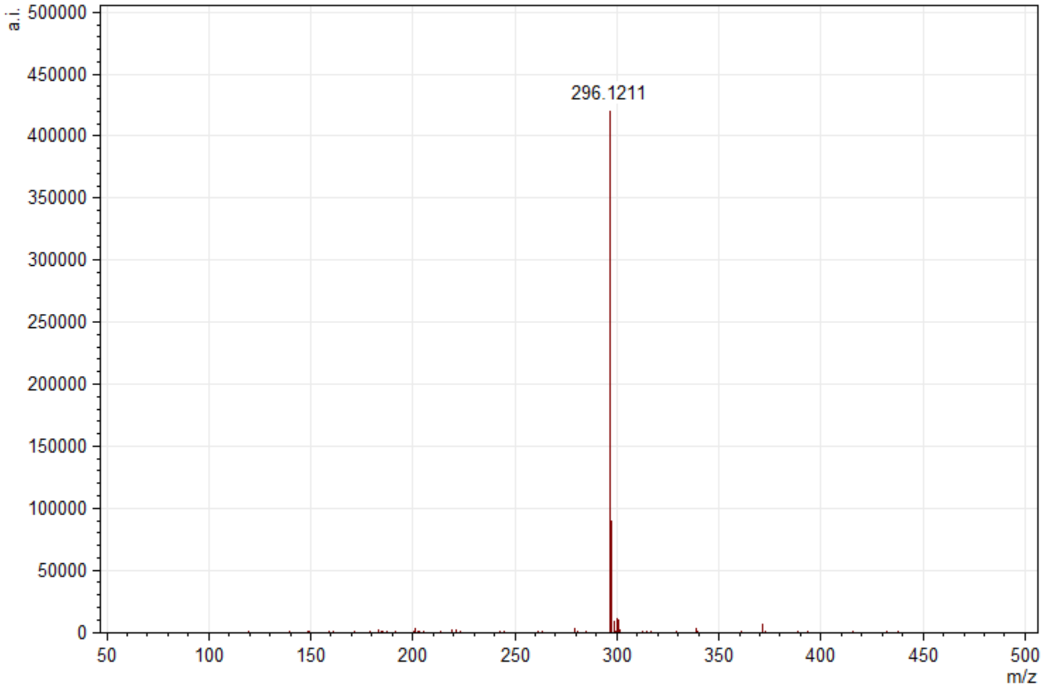

Figure S22 - HRMS (ESI) of compound **5b**

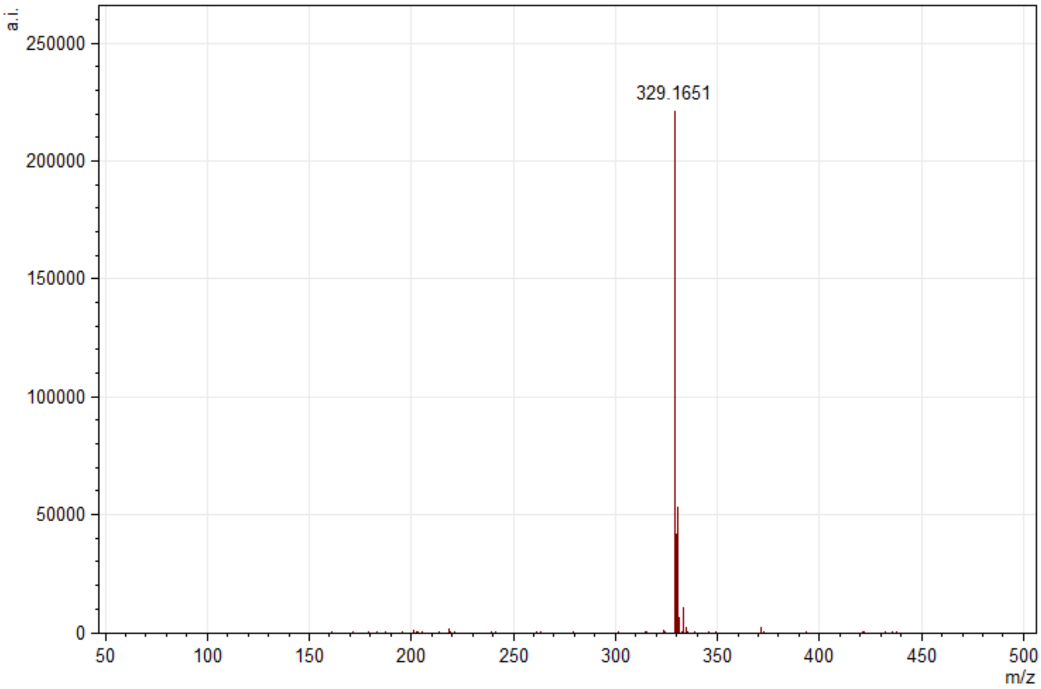

Figure S23 - HRMS (ESI) of compound **5c**

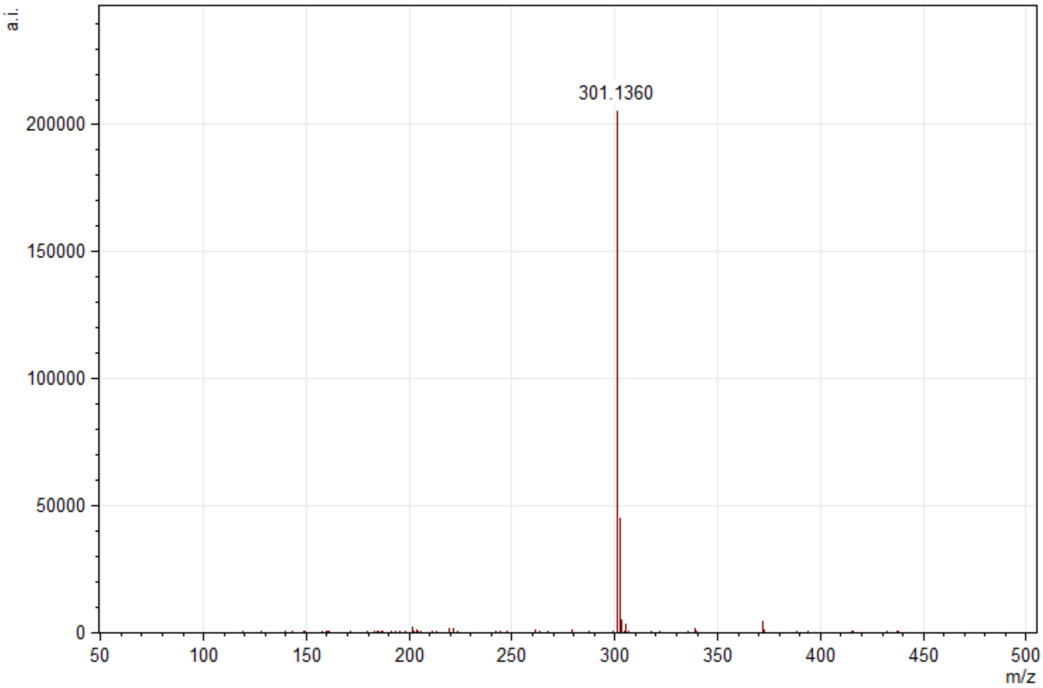

Figure S24 - HRMS (ESI) of compound **5d**

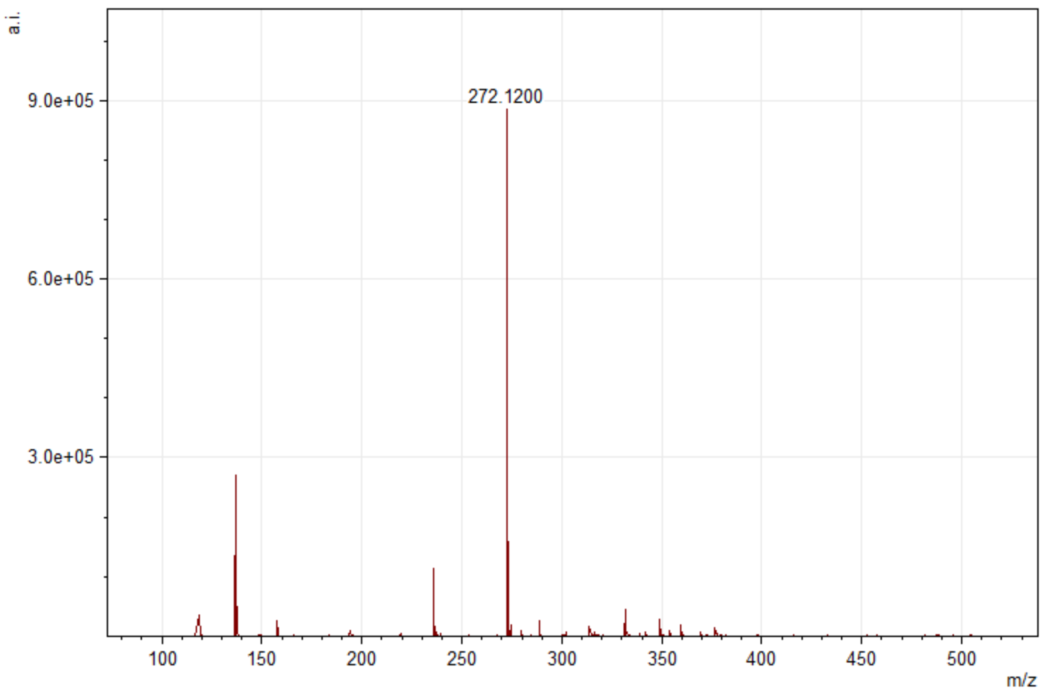

Figure S25 - HRMS (ESI) of compound **5e**

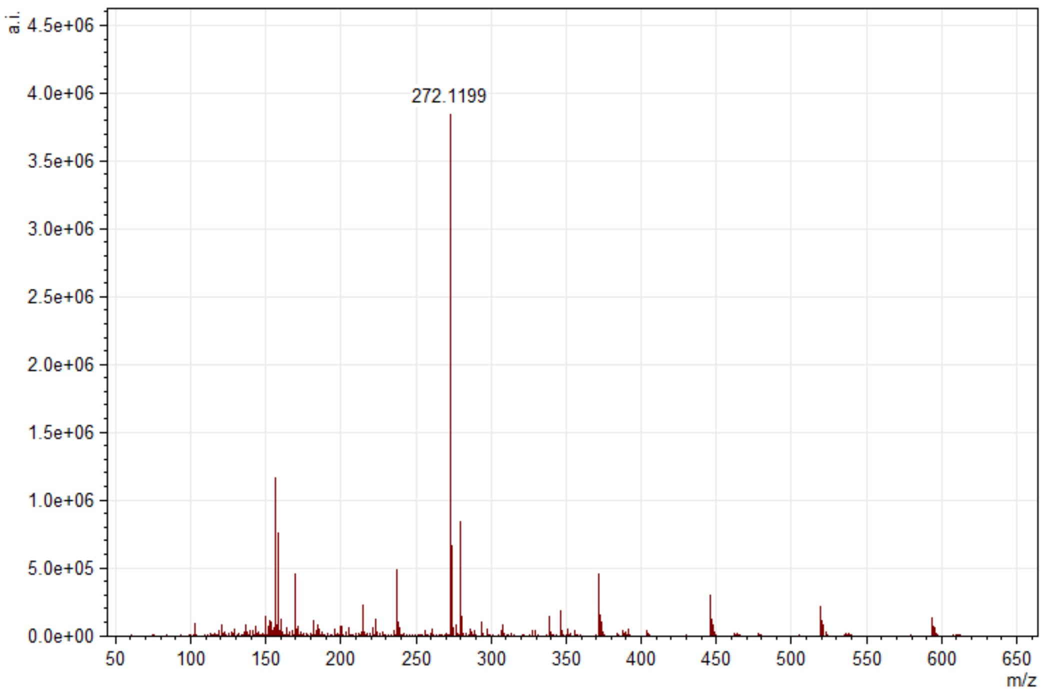

Figure S26 - HRMS (ESI) of compound **5f**

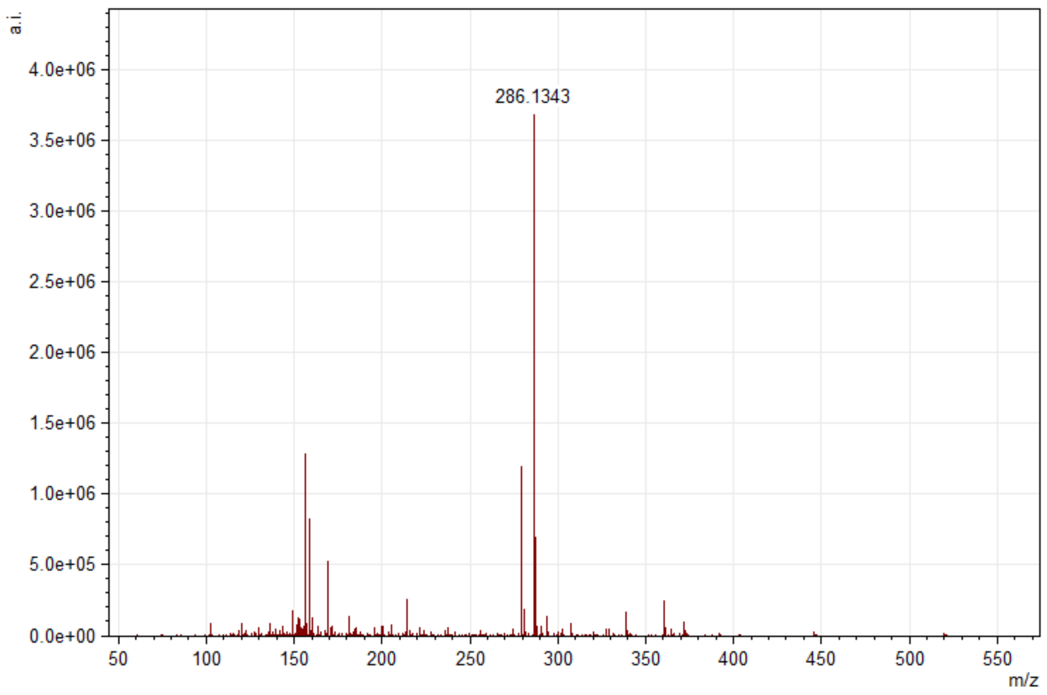

Figure S27 - HRMS (ESI) of compound **5g**

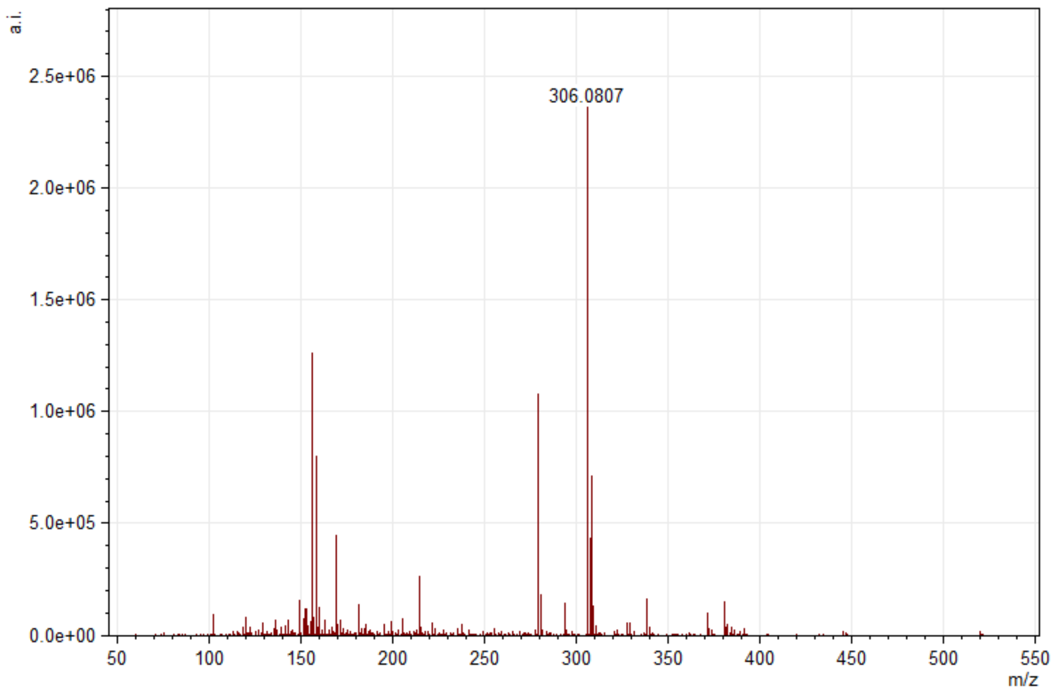

Figure S28 - HRMS (ESI) of compound **5h**

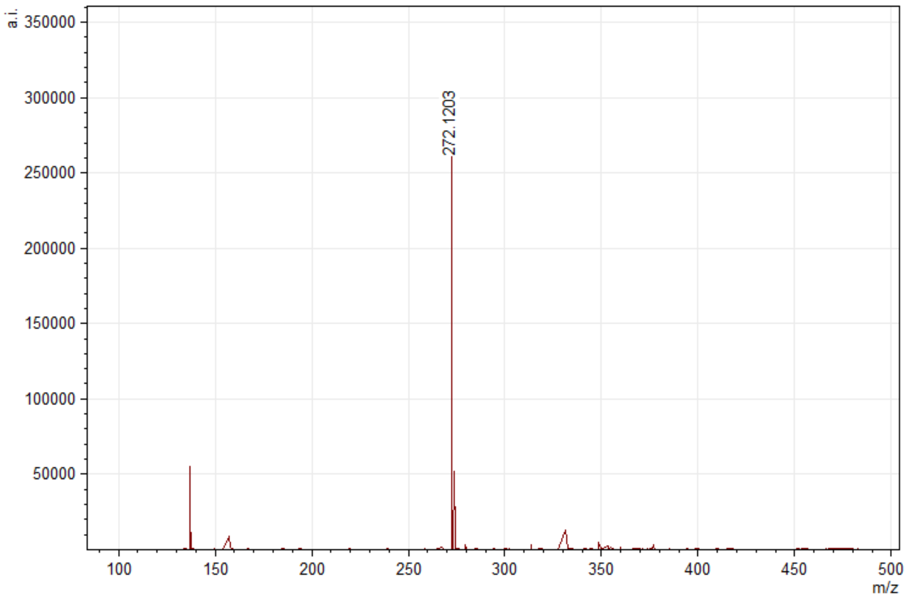

Figure S29 - HRMS (ESI) of compound **5i**

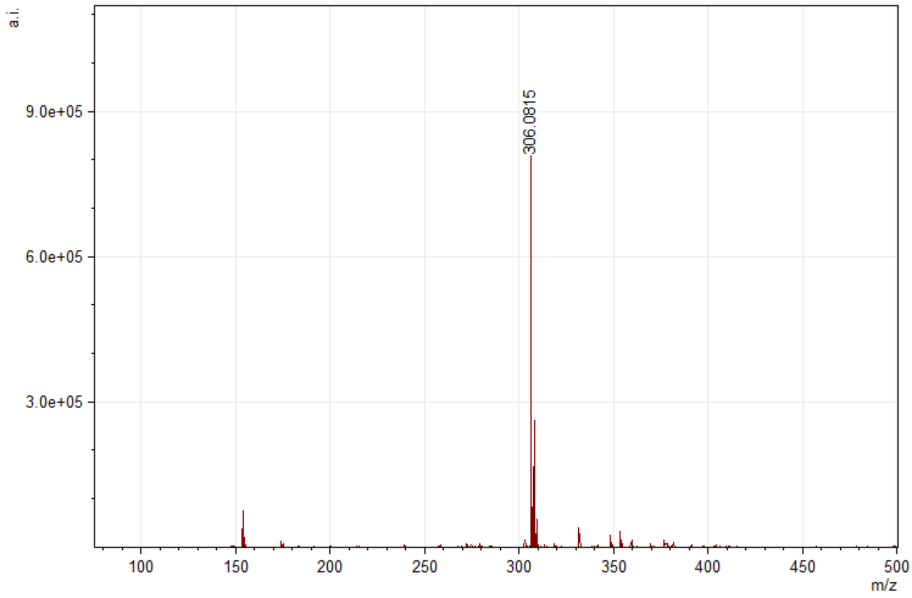

Figure S30 - HRMS (ESI) of compound **5j**

FTIR-ATR Spectra

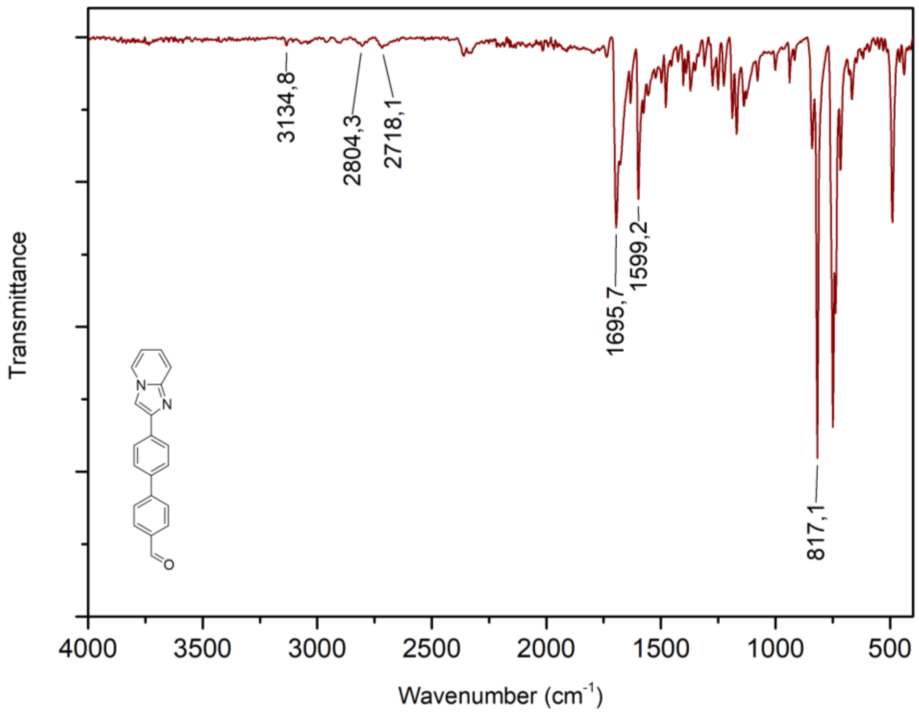

Figure S31 - FTIR-ATR spectrum of compound **5a**

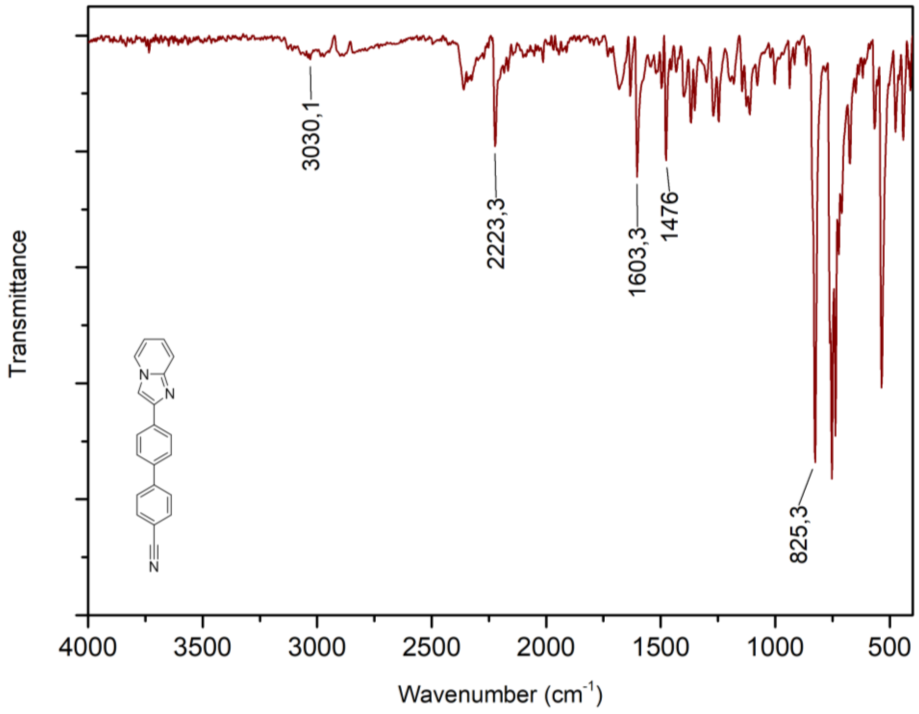

Figure S32 - FTIR-ATR spectrum of compound **5b**

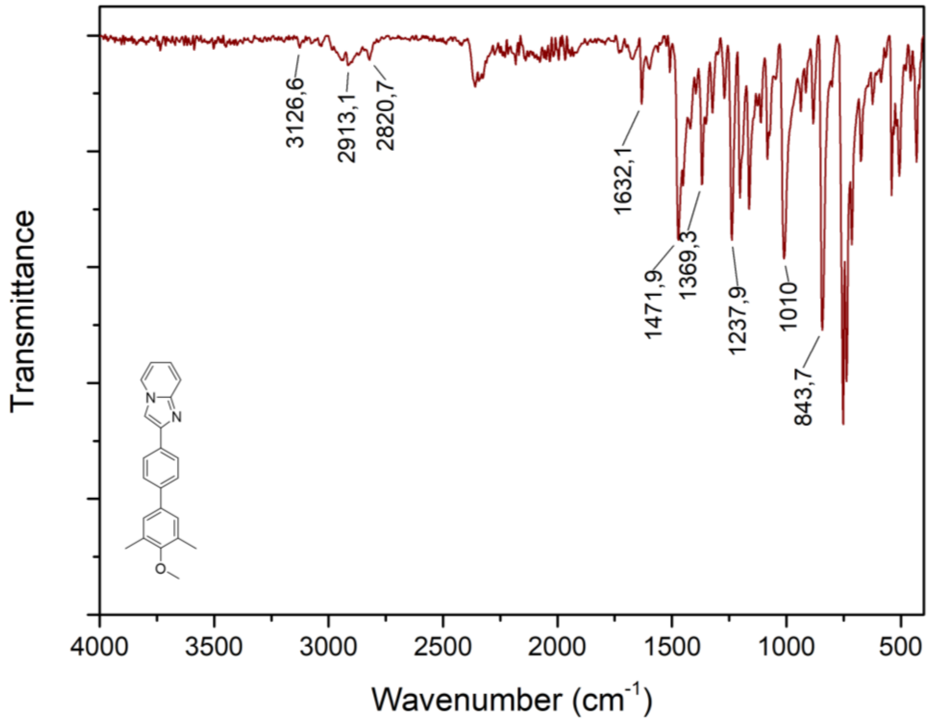

Figure S33 - FTIR-ATR spectrum of compound **5c**

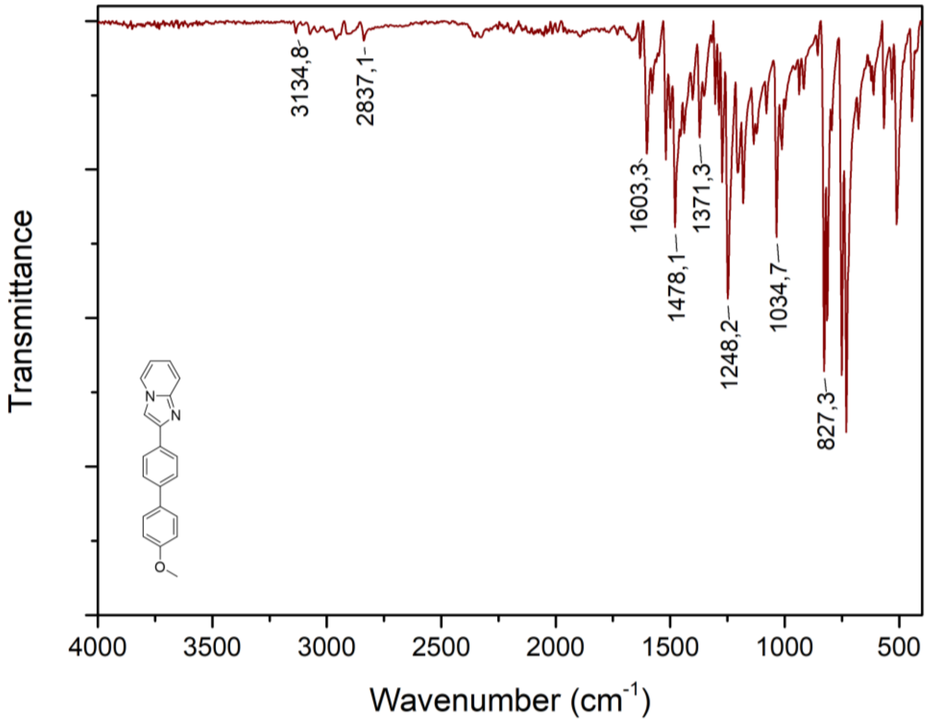

Figure S34 - FTIR-ATR spectrum of compound **5d**

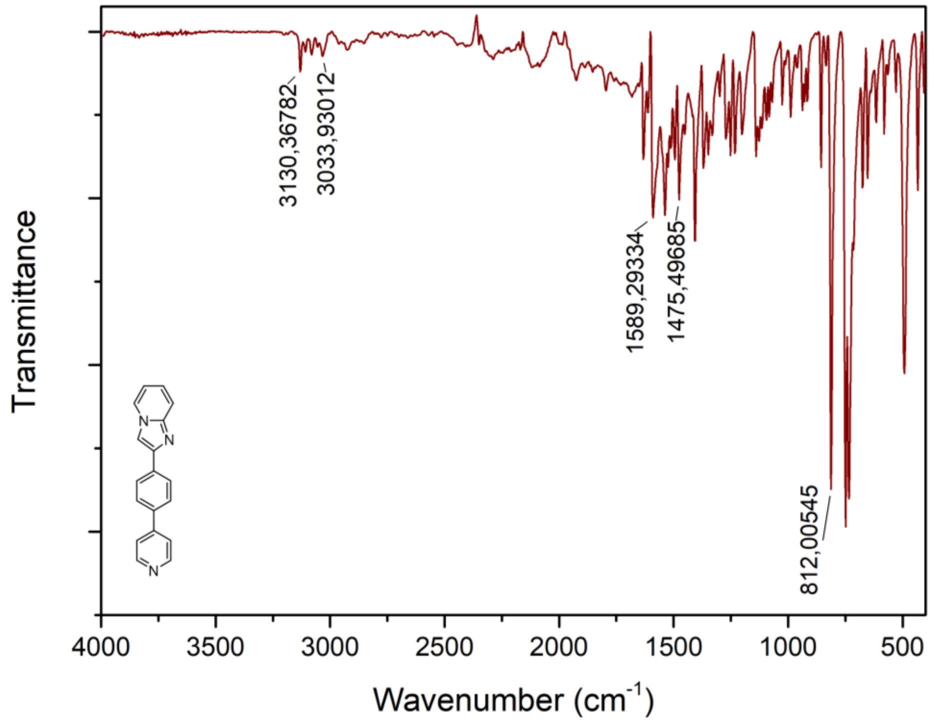

Figure S35 - FTIR-ATR spectrum of compound **5e**

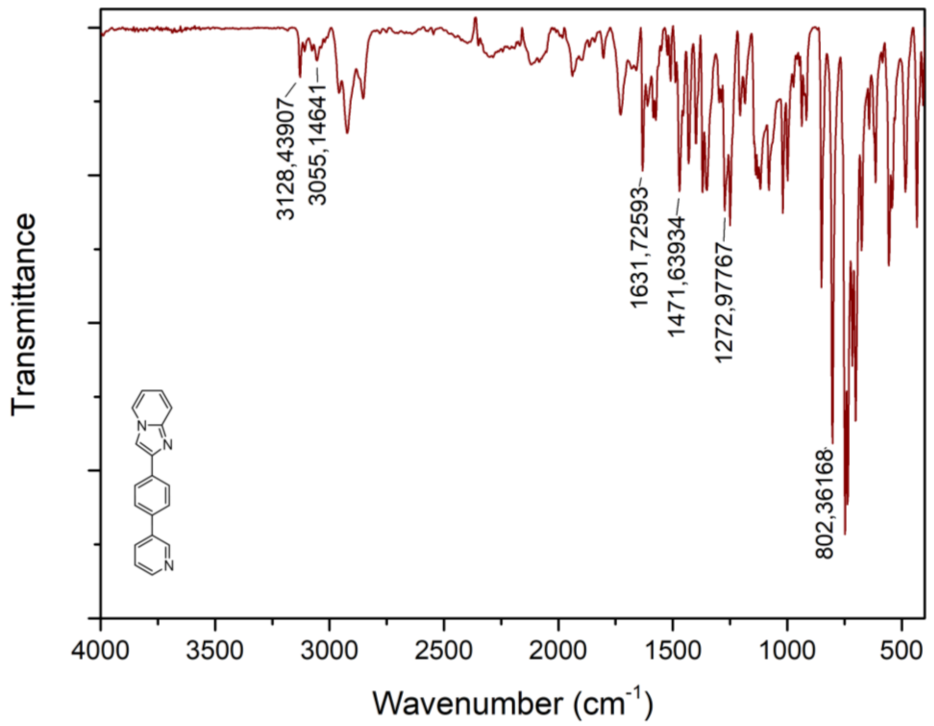

Figure S36 - FTIR-ATR spectrum of compound **5f**

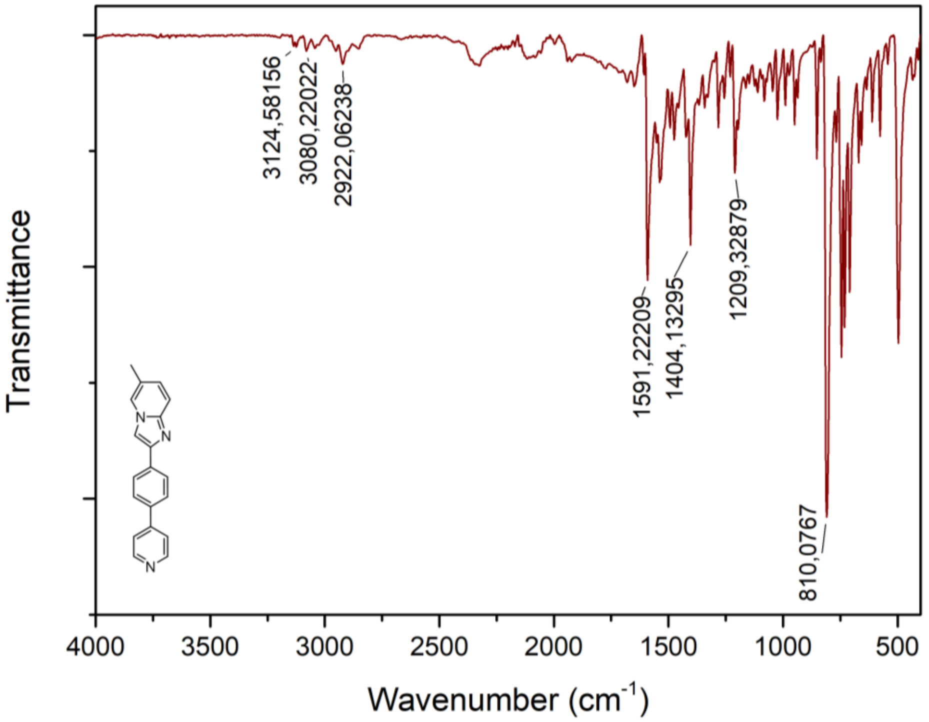

Figure S37 - FTIR-ATR spectrum of compound **5g**

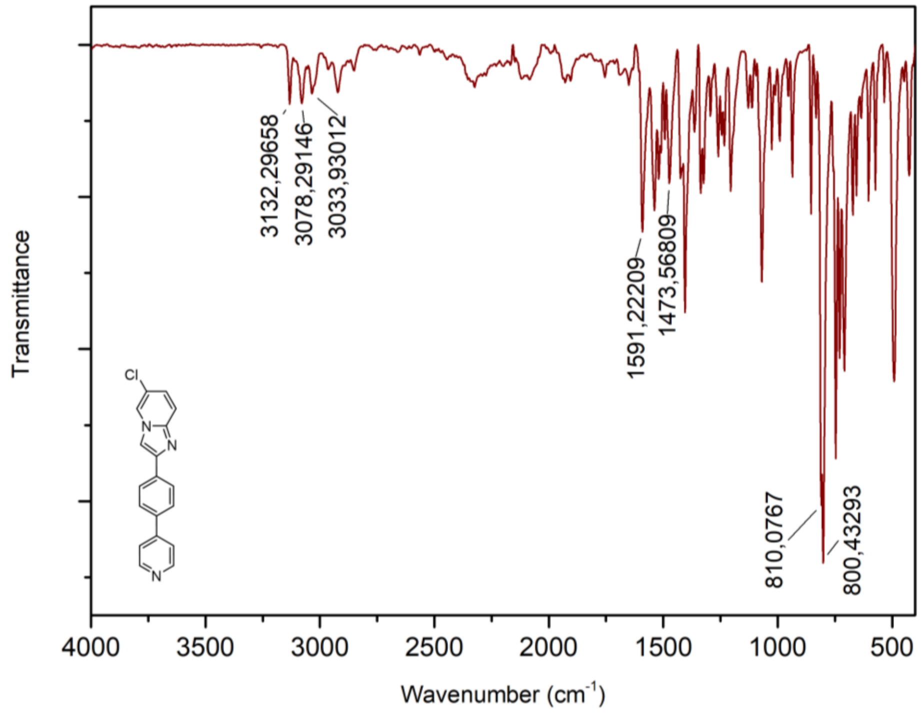

Figure S38 - FTIR-ATR spectrum of compound **5h**

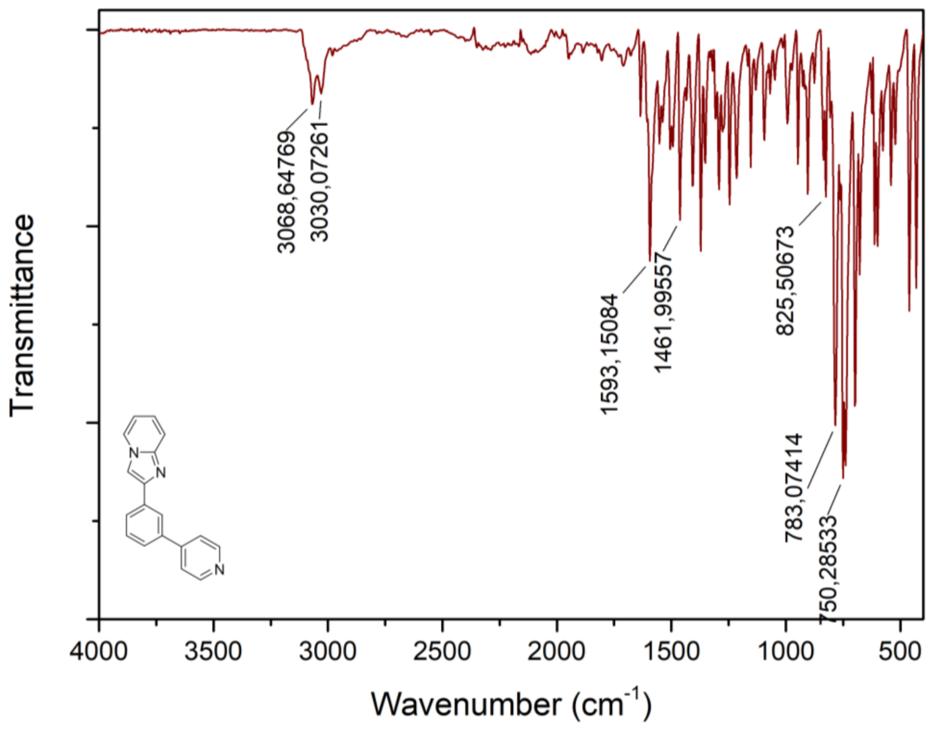

Figure S39 - FTIR-ATR spectrum of compound **5i**

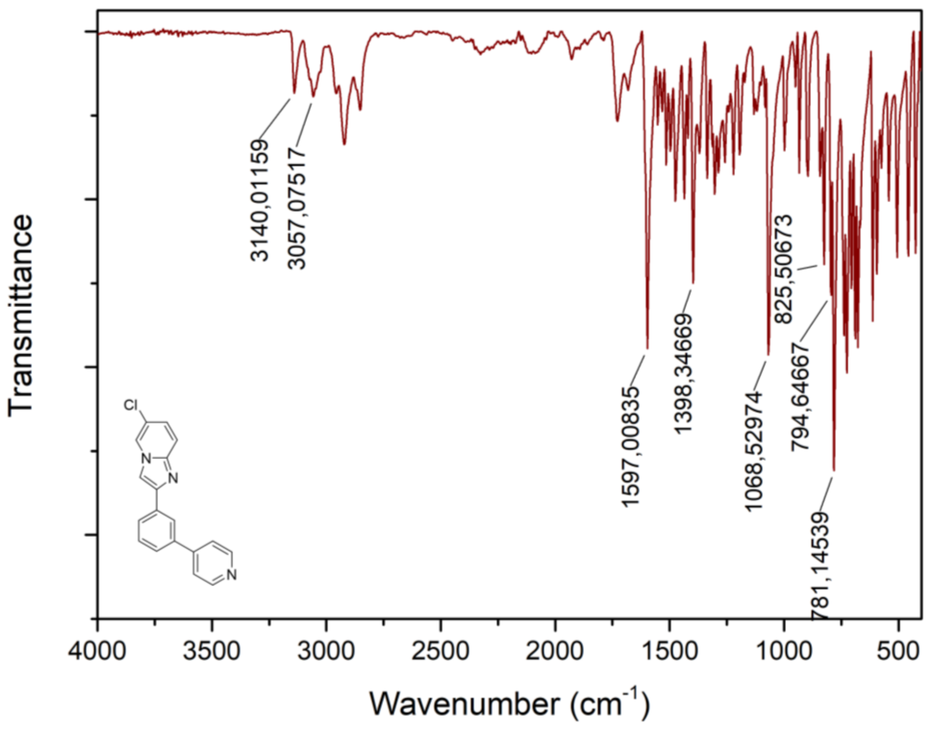

Figure S40 - FTIR-ATR spectrum of compound **5j**

Dose-response curves

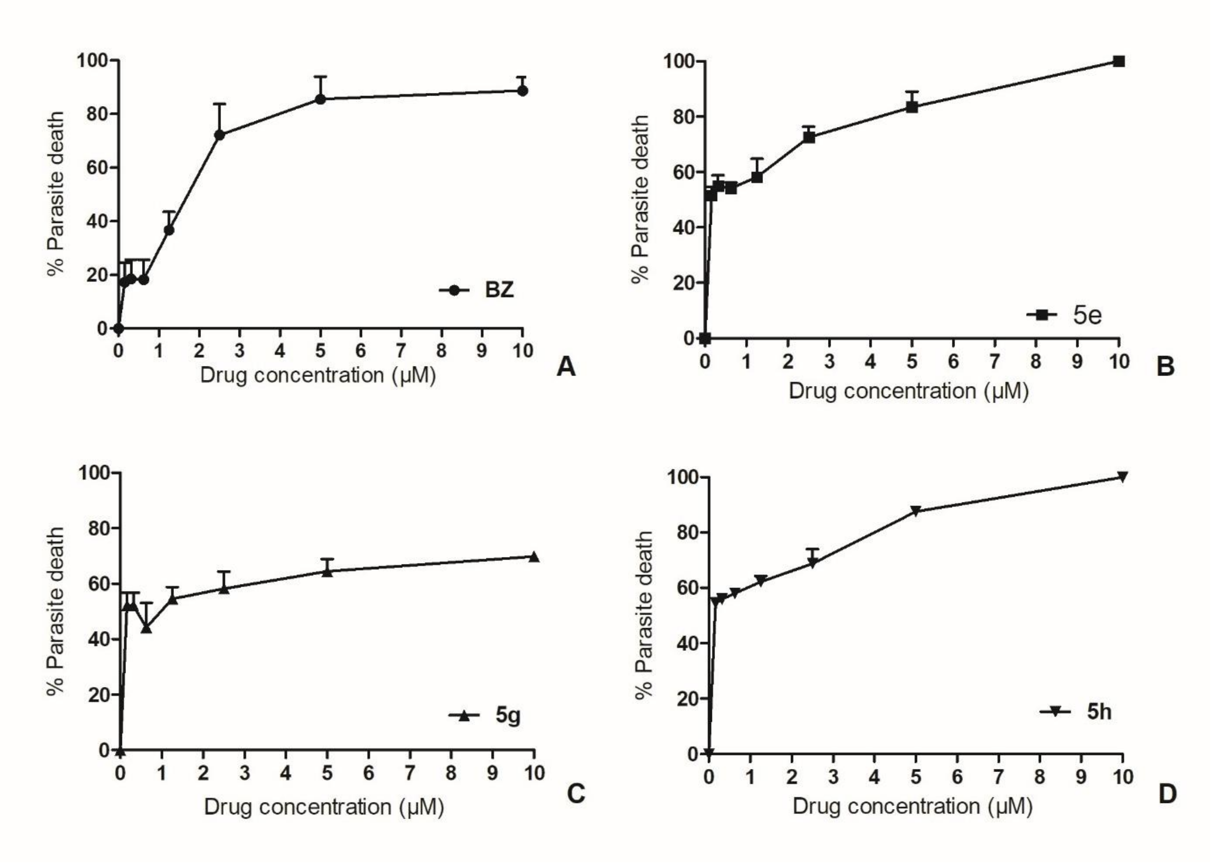

Figure S41 - Dose-response curves for the most active compounds **5e**, **5g** and **5h** along with Bz as reference drug.

Activity against cruzain

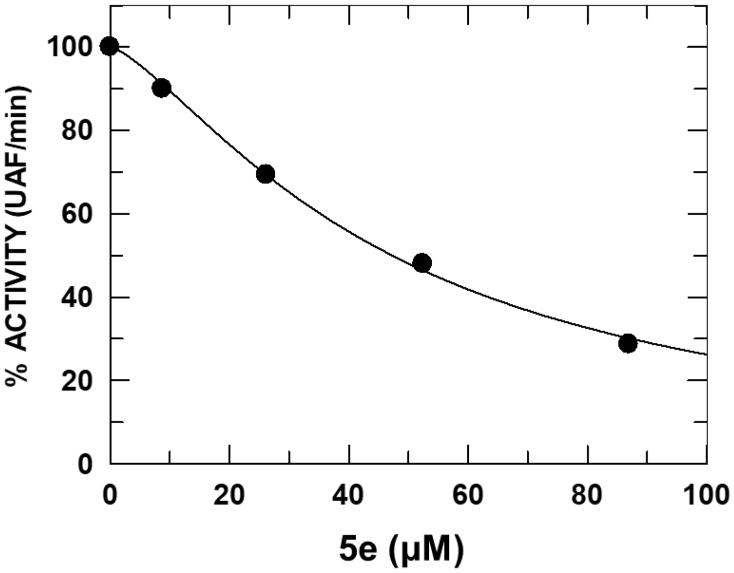

Figure S42 - Activity of compound **5e** against cruzain

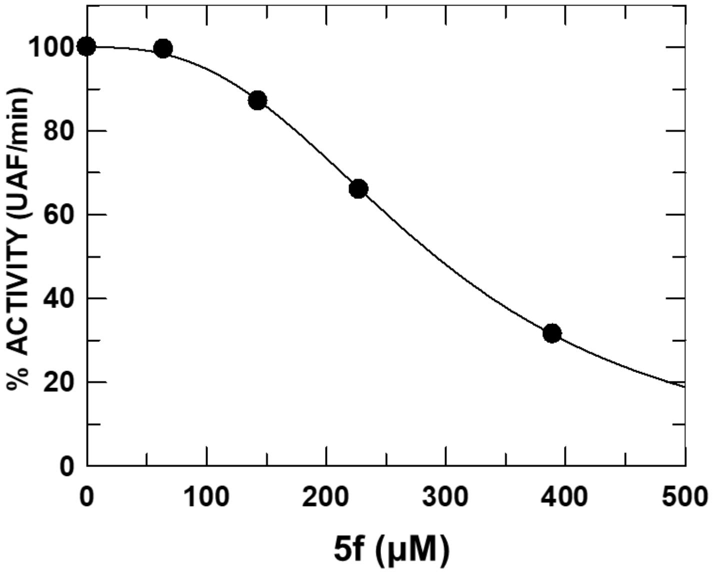

Figure S43 - Activity of compound **5f** against cruzain

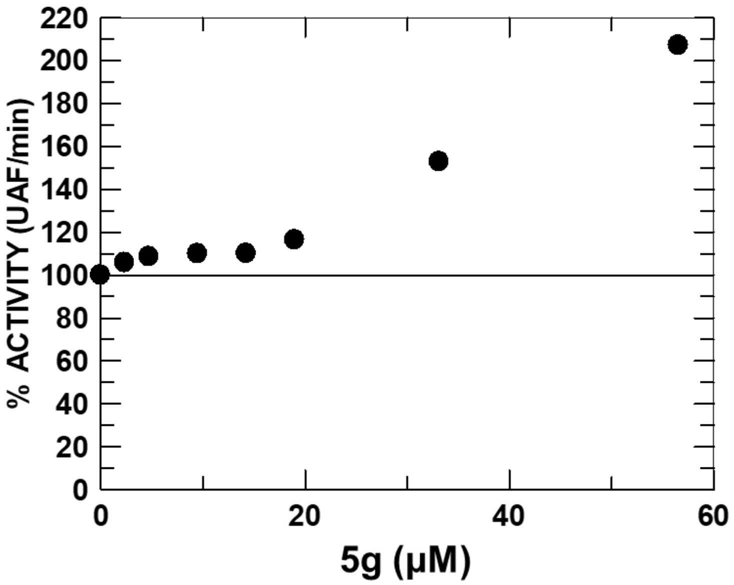

Figure S44 - Activity of compound **5g** against cruzain

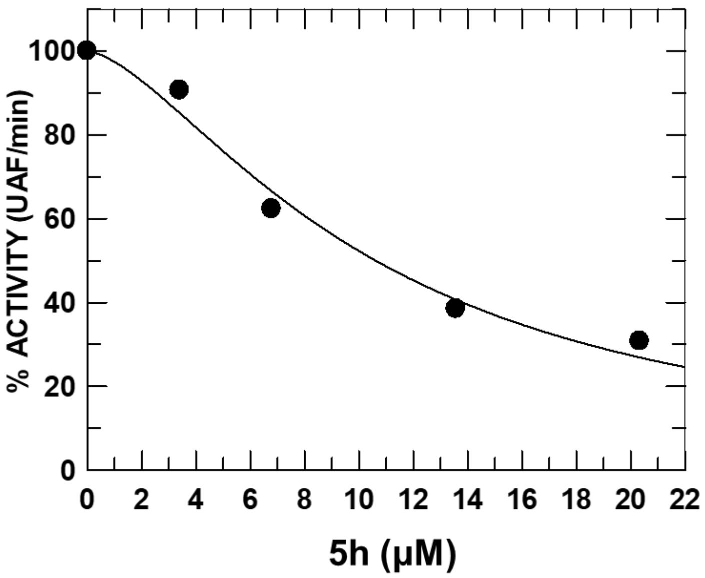

Figure S45 - Activity of compound **5h** against cruzain

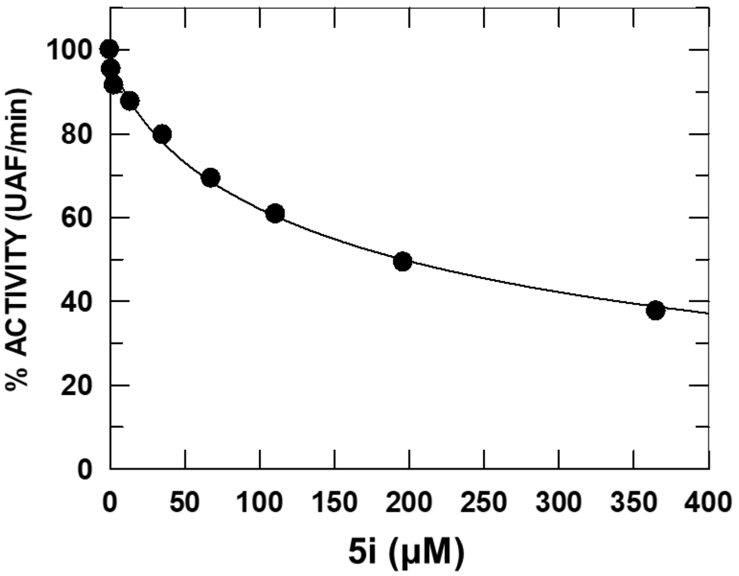

Figure S46 - Activity of compound **5i** against cruzain

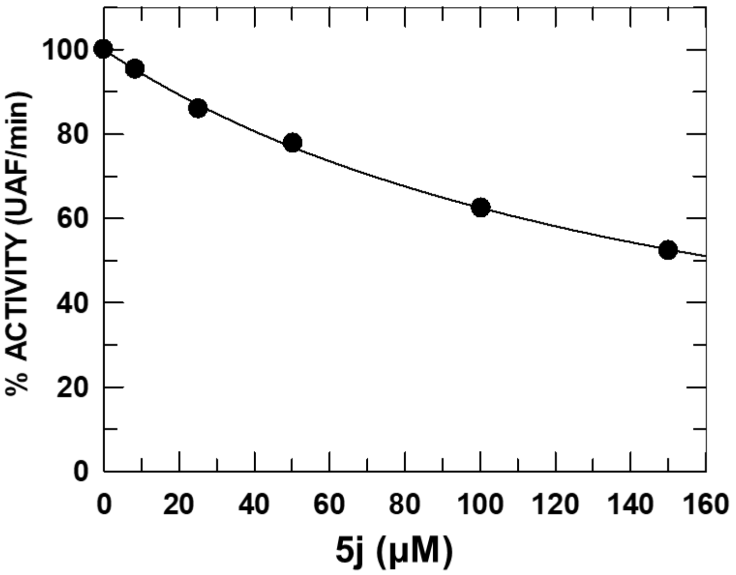

Figure S47 - Activity of compound **5j** against cruzain

Ramachandran plot for 1ME4

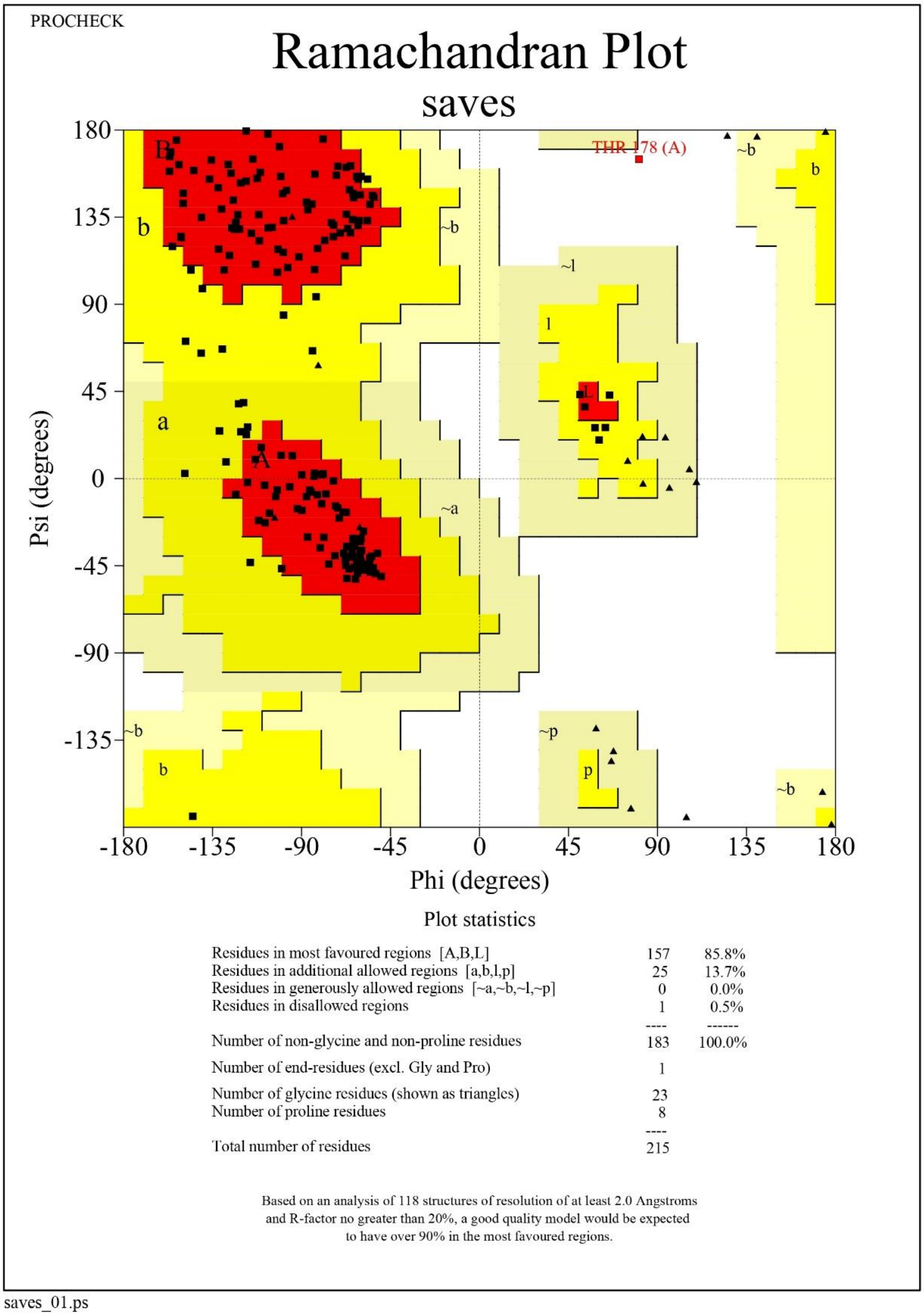

Figure S48 - Ramachandran plot for cruzain structure (PBD ID: 1ME4).

Table S1. Docking scores obtained for the evaluated compounds.

Compound	Scores
5e	50.2063
5f	50.1299
5g	48.4861
5h	51.6473
5i	51.2259
5j	53.5577

Table S2. Key amino acid residues involved in the binding of compounds **5e-j**.

Compound	Classical H-bond	Non-classical H-bond	π-Alkyl	π-Anion	π-Cation	π-σ	π-Sulfur	Alkyl	Halogen
**5e**			Leu67, Met68 and Ala133		Glu205		Cys25 and Met68		
**5f**	Asn69	Glu205	Met68 and Ala133	Glu112	Glu205	Leu67	Cys25 and Met68		
**5g**		Glu205 and Asn69	Ala133 and Leu67	Glu112	Glu205		Cys25 and Met68		
**5h**	Asn69	Glu205	Met68 and Ala133	Glu112	Glu205	Leu67	Cys25 and Met68		
**5i**		Gly66 and Asp158	Cys25, Leu67, Ala133 and Leu157				Met68		
**5j**		Gly66 and Asp158	Cys25, Trp26, Leu67, Ala133 and Leu157		Glu205		Met68	Cys25	Gly23
